# Automated detection and manipulation of sleep in *C. elegans* reveals depolarization of a sleep-active neuron during mechanical stimulation-induced sleep deprivation

**DOI:** 10.1038/s41598-018-28095-5

**Published:** 2018-06-27

**Authors:** Jan Spies, Henrik Bringmann

**Affiliations:** Max Planck Institute for Biophysical Chemistry, Am Fassberg 11, 37077 Göttingen, Germany

## Abstract

Across species, sleep is characterized by a complex architecture. Sleep deprivation is a classic method to study the consequences of sleep loss, which include alterations in the activity of sleep circuits and detrimental consequences on well being. Automating the observation and manipulation of sleep is advantageous to study its regulation and functions. *Caenorhabditis elegans* shows sleep behavior similar to other animals that have a nervous system. However, a method for real-time automatic sleep detection that allows sleep-specific manipulations has not been established for this model animal. Also, our understanding of how sleep deprivation affects sleep neurons in this system is incomplete. Here we describe a system for real-time automatic sleep detection of *C. elegans* grown in microfluidic devices based on a frame-subtraction algorithm using a dynamic threshold. As proof of principle for this setup, we used automated mechanical stimulation to perturb sleep behavior and followed the activity of the sleep-active RIS neuron. We show that our system can automatically detect sleep bouts and deprive worms of sleep. We found that mechanical stimulation generally leads to the activation of the sleep-active RIS neuron, and this stimulation-induced RIS depolarization is most prominent during sleep deprivation.

## Introduction

Sleep is a behavioral and physiological state that is found in all animals that have a nervous system and that have been studied carefully. It is defined by behavioral criteria such as the absence of voluntary movement, an increased arousal threshold, and homeostatic regulation^1,2^. Across species, sleep is induced by so-called sleep-active neurons, which are interneurons that are activated at the onset of sleep and actively induce sleep by inhibiting wake circuits through GABA and neuropeptides^3^. Depolarization of sleep-active neurons is believed to be a signature of sleep pressure because sleep deprivation leads to an increase of sleep-active neuron depolarization. However, how neural circuits control the activity of sleep-active neurons is not known.

Sleep is believed to be essential for animal life because it is widespread, under homeostatic regulation, which prevents sleep from being skipped, and because its deprivation typically has devastating consequences^2^. Stimulating an animal to prevent it from sleeping or to wake it up again are typical procedures to deprive an animal or human of sleep^4^. Sleep deprivation is used to study the regulation of sleep by looking at the increased sleep drive underlying sleep homeostasis and can also be used to study the consequences of sleep loss on wellbeing. Sleep deprivation has wide physiological consequences including impaired memory and immune function^5,6^. Severe sleep deprivation through sensory stimulation is inherently stressful and can lead to death in both vertebrates and invertebrates^4,7,8^. However, it is often not clear to what extent the phenotypes observed after sleep deprivation are caused by stress as opposed to the loss of sleep. Also, sleep deprivation increases sleep drive and how this increased sleep drive contributes to the phenotypes of sleep loss is often difficult to disentangle. Thus, it is important to be able to control sleep deprivation and to monitor its consequences for the nervous system.

*C. elegans* is an important model system for molecular neurobiology with a short generation time of about three days under ideal conditions, a small and invariant nervous system consisting of 302 neurons in the hermaphrodite with a known connectivity^9,10^. *C. elegans* is transparent allowing non-invasive imaging and optogenetics^11,12^.

Like other animals that have a nervous system, *C. elegans* also sleeps. While behavioral quiescence in “the worm” has been known for a long time, only recently has it been proposed that *C. elegans* actually experiences a sleep state as defined by behavioral criteria that include decreased voluntary movement, increased arousal thresholds, a relaxed posture, and homeostatic regulation^13–18^. Subsequent mechanistic and molecular dissection of sleep in *C. elegans* has demonstrated similarities to sleep in other animals, suggesting that sleep has a common evolutionary origin across species^8,19–25^.

Worms are behaviorally quiescent in the larva during development and in the adult after cellular stress or under various food conditions including satiety and starvation. Among the best-understood types of sleep in *C. elegans* in mechanistic terms is developmentally controlled sleep^26–28^. *C. elegans* larvae show sleeping behavior at the end of each of the four larval cycles during a developmental stage and behavioral state called lethargus, which is characterized by behavioral inactivity^13^. During lethargus, worms stop feeding and show reduced locomotion characterized by bouts of complete immobility^13–15^. Lethargus coincides with the phase during which the animals synthetize a new cuticle and before the old cuticle is shed^29^. Sleep bouts during lethargus are induced by a single sleep-active neuron called RIS. This neuron is most active at the onset of sleep, it can induce quiescence if activated optogenetically, and is crucially required for sleep bouts^21^. RIS expresses GABA and the inhibitory FLP-11 neuropeptides that are required for sleep induction^25^. Thus, RIS presents a *bona fide* sleep-active neuron with high similarities to its mammalian counterparts. As in mammals, disturbing sleep in *C. elegans* can lead to an activation of RIS^25,30,31^.

Sleep deprivation by mechanical stimulation has been shown to be stressful in *C. elegans* and stress-sensitive mutants can even be killed by prodding the worm during lethargus^8^. More gentle stimulation by dish tapping can be used to wake up the animal and if performed repeatedly it can activate stress responses and causes modest phenotypes, most prominently in stress-sensitive mutant backgrounds^32,33^. Genetic ablation of sleep generally produces much weaker phenotypes during lethargus: Deletion of the *aptf-1* transcription factor gene results in viable mutants but completely abolishes sleep bouts. This suggests that mechanical stimulation during sleep causes lethality independently of sleep loss^21,34^.

Sleep deprivation has been performed either manually or by using a stereotyped protocol on synchronized populations^8,32^. However, a system that automatically detects and removes sleep has not been established for *C. elegans*. Such a system would reduce tedious and time-consuming manual procedures and would reduce bias introduced by an experimenter. Also, it would provide precision that would not be achievable by synchronizing animals and would allow state-specific experiments. Here we describe a method for automated real time recognition of sleep behavior in *C. elegans* growing in microfluidic chambers. Continuous DIC imaging allows detection of sleep through image subtraction using a dynamic threshold implemented as a Labview routine. Once sleep is detected, it can be automatically reversed by providing a mechanical stimulus by tapping the dish that contains the microfluidic device. The system allows simultaneous calcium imaging. We demonstrate the capacity of this system to deprive *C. elegans* of sleep and to monitor the activity of the RIS neuron. Mechanical stimulation always appears to trigger RIS depolarization and this effect is most pronounced during lethargus. Thus, RIS activity appears to be the product of mechanical stimulation and homeostatic sleep pressure induced by sleep deprivation.

## Results

### A setup for automated sleep detection, state-specific stimulus delivery, and calcium imaging

**Figure 1:**
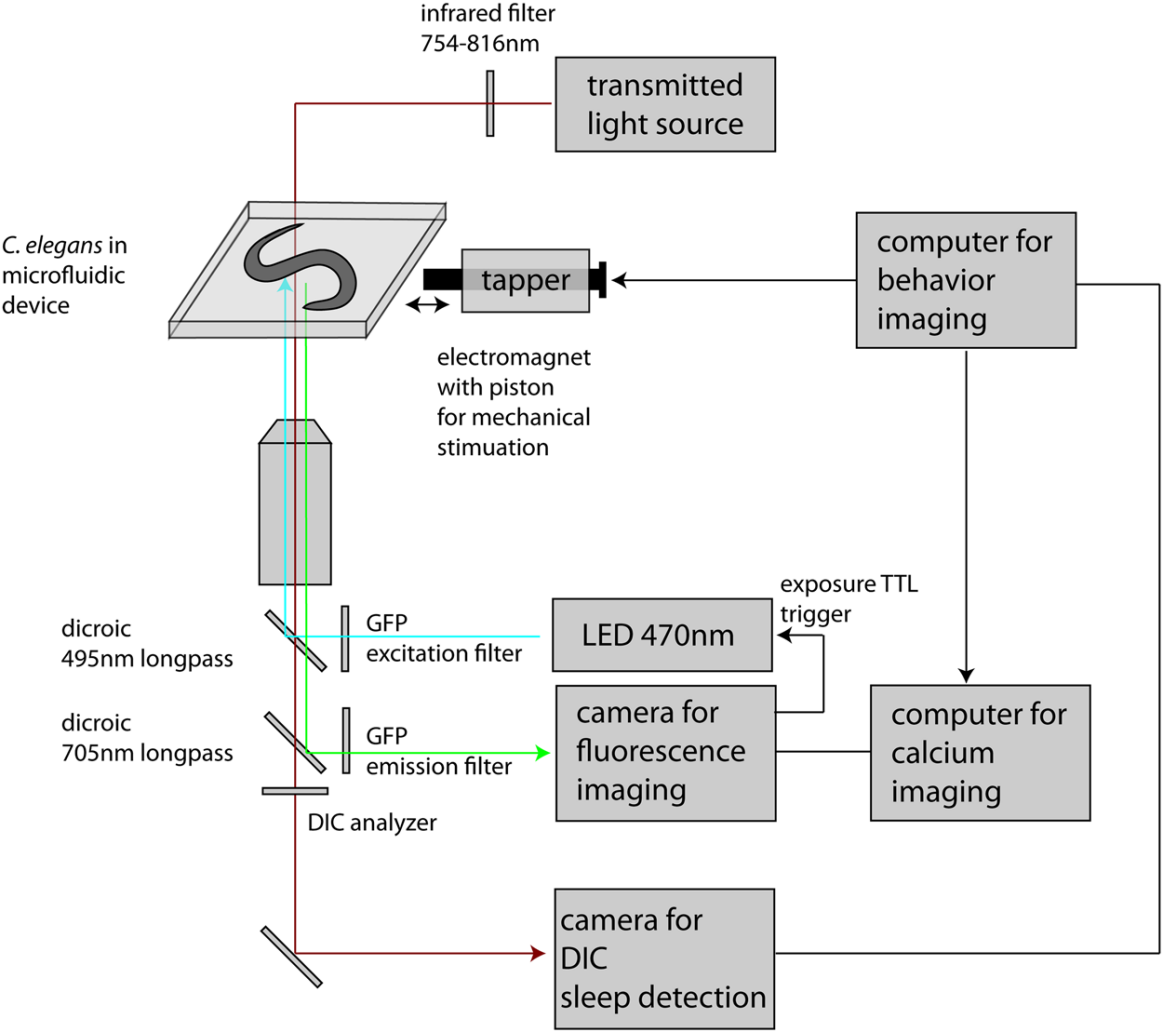
Scheme of the setup for automatic sleep detection and deprivation. Worms are kept inside microfluidic devices. The system allows continuous filming with DIC using transmitted infrared light and simultaneous fluorescence imaging. The DIC camera detects sleep using a frame subtraction algorithm using a dynamic threshold. We deprived the worm of sleep by dish tapping.

Automated real-time detection would allow unbiased state-specific experiments, but this has not been established for the study of sleep in *C. elegans*. We thus aimed to develop a system that is able to automatically monitor in real time the behavioral state of *C. elegans*, deliver a state-specific stimulus, such as a mechanical impetus, and record fluorescence calcium imaging data for the analysis of neuronal activity. To build a setup for simultaneous behavioral imaging and calcium imaging we used a microscope with transmitted light and with two layers of filter wheels. We used transmitted infrared light for DIC imaging of behavior onto the side port of the microscope, where a first camera was installed and controlled by a first computer. To allow fluorescence microscopy, we used a dichroic mirror to reflect blue light from a LED passing through the top layer filter wheel. We imaged fluorescence through the bottom layer filter wheel using the back port of the microscope where a second camera was installed that was controlled by a second computer (see Fig. 1 for a schematic and Methods for detail). This system thus allowed a continuous imaging of behavior using transmitted infrared light-based DIC and fluorescence calcium imaging.

To keep the worm in the field of view of the cameras it was placed inside a microfluidic chamber made from hydrogel^35,36^. Briefly, casting agarose onto a PDMS mold generated a shallow box-shaped indentation. The indentation was then filled with bacterial food and a worm egg. The chamber was then sealed with a glass slide. After hatching, the L1 worm was trapped inside the chamber where it was observed through L1 lethargus until the L2 stage.

We wrote a program for automated detection and deprivation of sleep using Labview. In this software integration of interfaces for many devices, such as cameras and data acquisition cards is preexisting and a library exists containing functions for data acquisition, mathematics, statistics, and an advanced graphical user interface (GUI). Because Labview allows a graphical programming approach, it will be easier for future users that do not have a background in programming to modify the program according to their needs.

**Figure 2:**
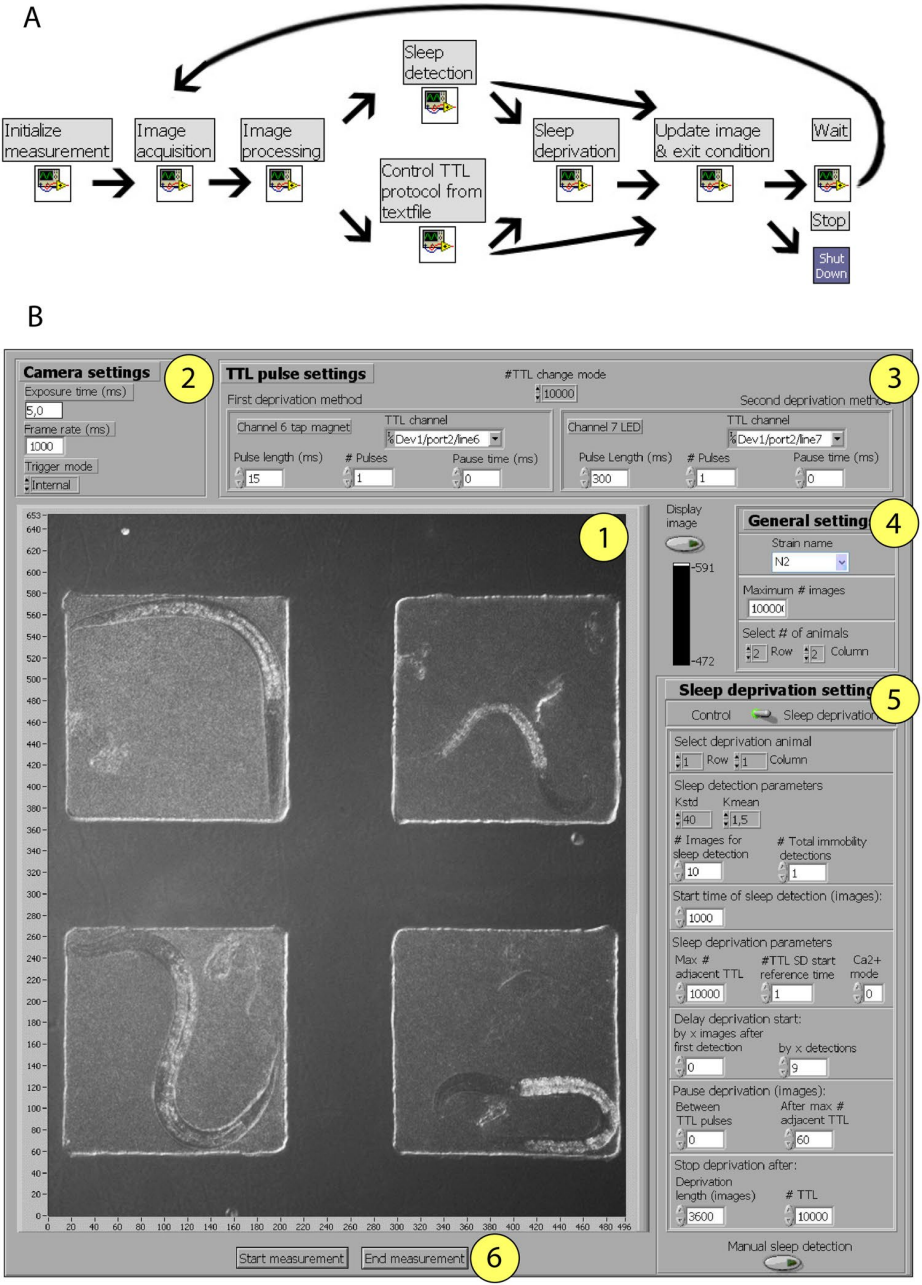
Algorithm and computer interface for automatic sleep detection and deprivation. (**A**) Labview program scheme (**B**) Labview GUI Control window. Key elements are labeled with numbers: (1) Display of camera image, (2) Camera acquisition settings, (3) TTL-pulse settings, (4) General settings, (5) Sleep deprivation settings, (6) Start or End measurement.

Labview functions are called virtual instruments (VIs). Instead of a written source code each VI consists of a block diagram, a front panel, and a connector panel. The block diagram is the graphical equivalent of the written source code. The front panel is a graphical user interface corresponding to the block diagram that is automatically created by Labview. The connector panel represents the input and output parameters of the VI that have to be used by another VI to call this VI. The “*C. elegans* automated sleep deprivation” program is based on the call of nine subroutines and is controlled by a GUI (Fig. 2). In this program a measurement always starts with the “Initialize measurement VI”, that is followed by the “Image acquisition VI” and the “Image processing VI”. These modules control the imaging parameters. Depending on whether the sleep deprivation or the control mode is chosen, either the “Sleep detection VI” or the “Control TTL protocol from textfile VI” is executed. Sleep detection is achieved via image subtraction, which is an established method for detecting sleep bouts^14,15,20,37,38^. In case of successful sleep detection, “Sleep deprivation VI” follows, or in case of no sleep detection the program continues directly with “Update image detection & exit condition VI”. Sleep deprivation is carried out through mechanical stimulation. Evaluation of the exit condition either runs the “Wait VI” or shuts down the measurement with the “ShutDown VI”. The “Wait VI” then triggers again the “Image acquisition VI”, leading to a cyclic VI execution. A detailed annotation of the source code and a description of setting up the parameters is provided in the supplement (supplemental file describing the “*C. elegans* automated sleep deprivation” program). All files required for running the program are available as supplementary data (supplementary source code folder). We used frame subtraction to detect sleep and used a dynamical threshold to robustly detect sleep across different strains and illumination conditions. Parameters were determined empirically based on a set of sample data. This algorithm was able to detect sleep in 89–96% of times as compared with a manually annotated data set (see Methods for details).

Dish-tapping is an established strategy to deliver gentle mechanical stimulation to *C. elegans*^18,33,39^. Mechanical stimulation by dish-tapping wakes up sleeping *C. elegans* during lethargus^32^. Because the microfluidic structures are mounted inside a plastic dish, a mechanical stimulus can be applied using an electromagnet that moves a piston to hit the dish. The resulting vibration is largely confined to the imaging plane and blurring of the image is prevented by staggering image acquisition and vibrational stimulus^18^.

As a general protocol we decided to deliver a mechanical tapping stimulus as soon as worms entered sleep bouts during lethargus to keep the worm in motion bouts. Whenever the worm returned to a sleep bout, an additional stimulus was given. This was repeated until the animal did not respond any longer to mechanical stimulation. Once stimulation no longer caused a detectable response, an extended stimulation protocol was run to monitor the effects of long-lasting stimulation.

**Figure 3:**
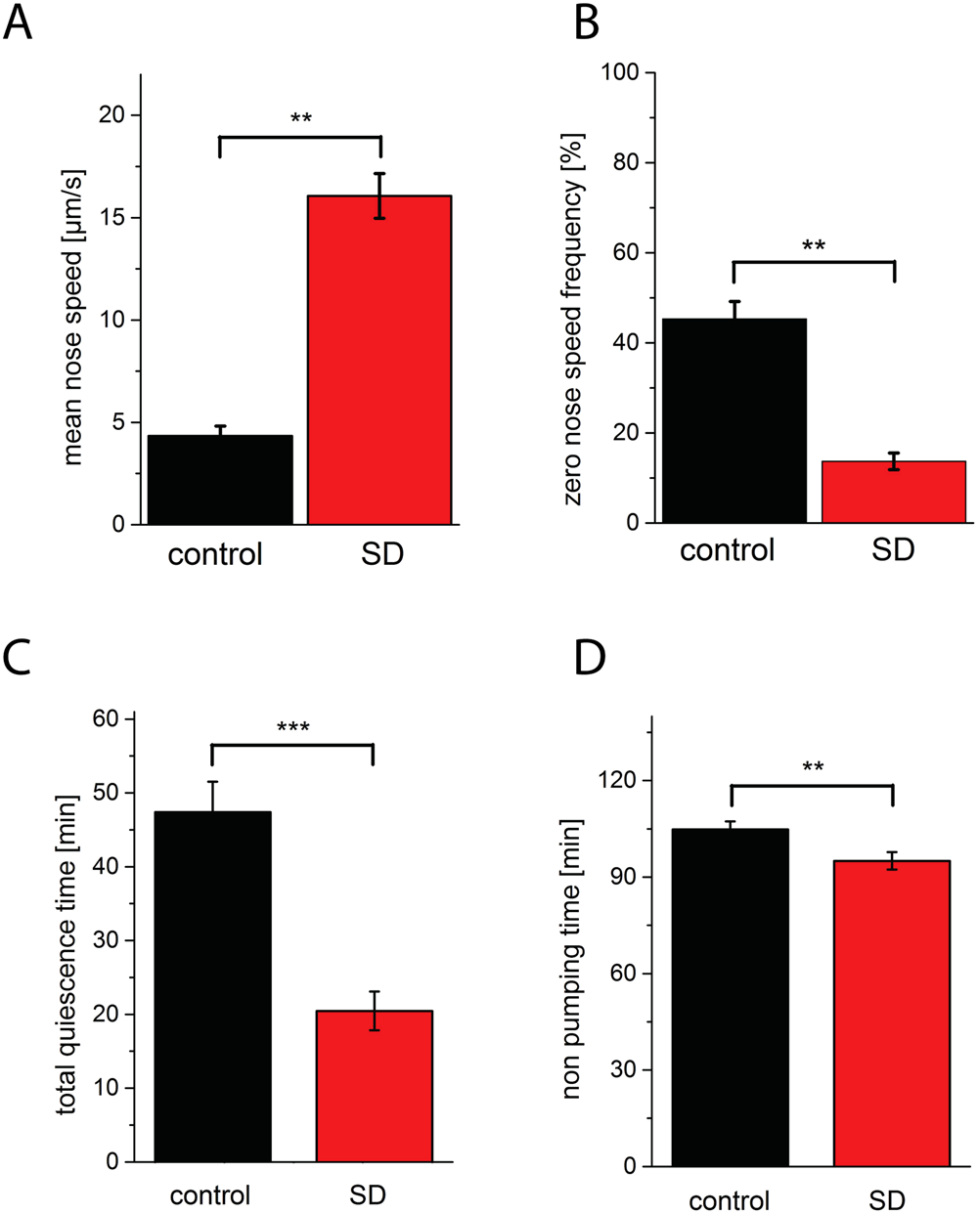
Sleep deprivation using dish tapping. (**A**) Dish tapping (sleep deprivation, SD) led to a 271% increase in mean mobility as measured by tracking nose speed. (**B**) Sleep bouts as defined by no detectable nose speed was reduced by 70% during the stimulation. (**C**) Total sleep time during L1 lethargus is reduced by 57% by sleep deprivation. (**D**) L1 lethargus length as measured by the time worms did not pump was slightly reduced by sleep deprivation by 9%. n = 9 worms, control animals received the tapping stimulus during L1 before lethargus. **Denotes statistical significance with p < 0.01, and ***denotes statistical significance with p < 0.001, paired Wilcoxon rank test.

In the GUI, one worm out of the four animals seen in the field of view was selected for sleep deprivation whereas one other worm, an individual, which was outside of lethargus during stimulation, was used as the control. Mechanical stimulation during sleep increased movement speed by more than two-fold to reach levels of activity that were similar to worms before entering lethargus of 16 μm/s (Fig. 3A). The frequency of sleep bouts, defined as zero (no detectable) nose movement, was reduced by 70% during the phase of mechanical stimulation (Fig. 3B). The time worms spent in sleep bouts during the entire phase of lethargus was decreased by 57% (Fig. 3C). To test whether sleep deprivation affected lethargus duration we determined the time the worms did not show peristaltic pumping behavior of the pharynx. Sleep deprivation shortened lethargus length slightly but significantly by 9%, suggesting that lethargus length is not extended homeostatically by sleep deprivation consistent with previous results^8^ (Fig. 3D). Thus, our system can not only automatically detect sleep but can also deny worms a significant fraction of sleep.

### Mechanical stimulation activates the sleep-active RIS neuron most strongly during lethargus

**Figure 4:**
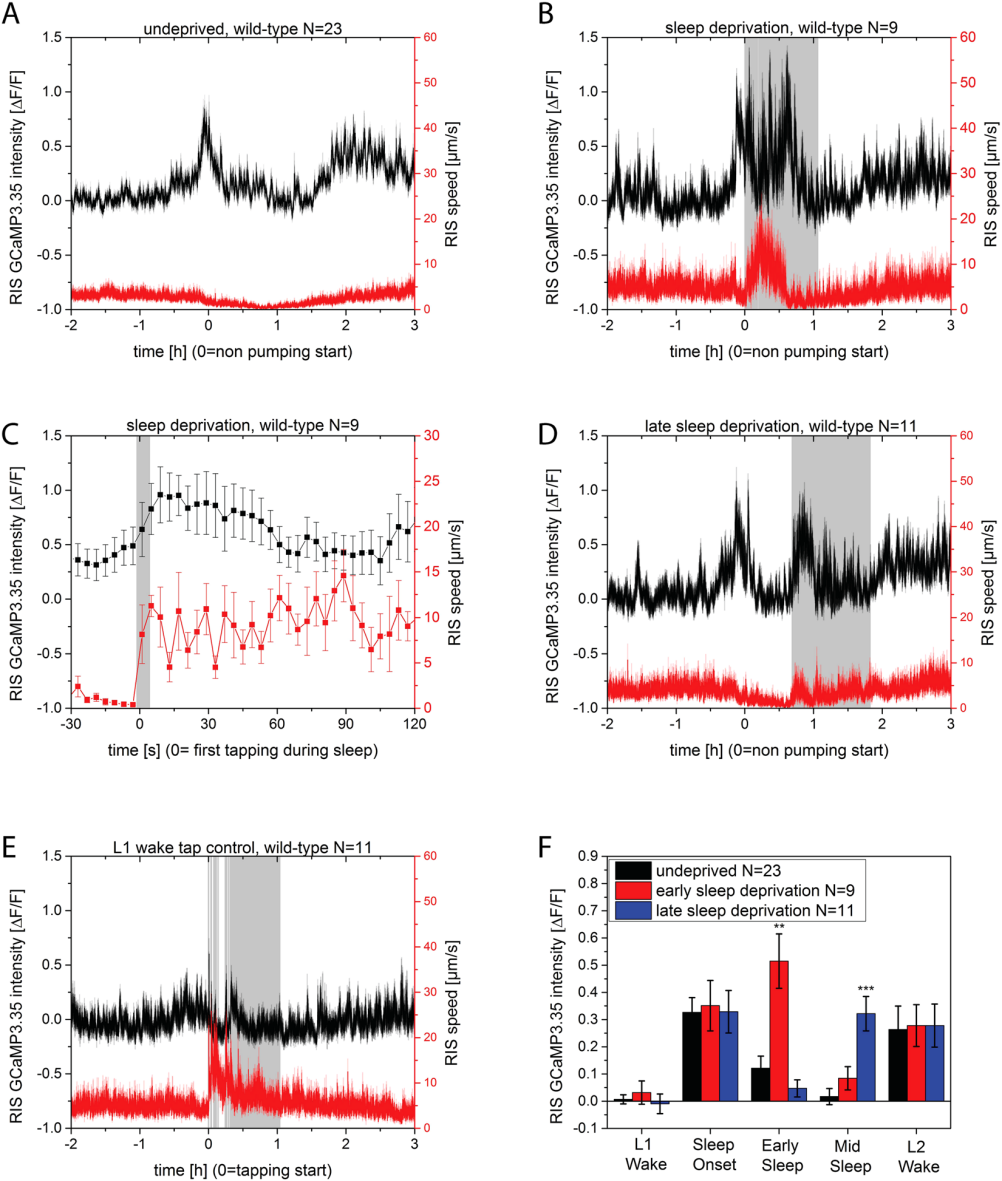
Mechanical stimulation increases RIS activity most strongly during lethargus. Sleep deprivation was applied using dish tapping. (**A**) Worms that do not get stimulated mechanically show two main phases during which RIS transients occur, the first during the onset of lethargus and one at the end of lethargus. Plot modified from Urmersbach *et al*., with permission (Wiley)^40^. (**B**) Sleep deprivation at the onset of sleep behavior leads to an additional activation phase of RIS, mostly during continuous tapping. (**C**) Extraction of the first response to mechanical stimulation shows a simultaneous increase in RIS activity and mobilization. (**D**) Sleep deprivation 30 minutes after the onset of sleeping behavior also led to an increase in RIS activation. (**E**) A comparable mechanical stimulation protocol outside of lethargus also leads to a small increase in RIS activation. (**F**) Quantification of RIS activation upon by measuring RIS GCaMP3 activity over 30 minutes before lethargus (“wake”), at the onset of lethargus (“sleep onset”, non-pumping phase start), early lethargus (“early sleep”), 30 minutes into lethargus (“mid sleep”), and after lethargus (“L2 wake”). **Denotes statistical significance with p < 0.01, and ***denotes statistical significance with p < 0.001, Welch test.

To monitor the activity of the sleep-active RIS neuron, we observed RIS activity using calcium imaging during the entire protocol. We expressed GCaMP3 from the *aptf-1* promoter, which expresses in AIB, RIB, and RIS^21^. The signal of RIS was extracted using an automated Matlab routine^40^. For averaging, data from individual worms were aligned to the start of lethargus defined as the non-pumping period. In non-stimulated control animals RIS showed increased activity at the onset of lethargus, which then decreased in the course of lethargus sleeping behavior, and activated again at the exit of lethargus (Fig. 4A)^21,40^. Sleep deprivation that started at the onset of lethargus led to increased mobility and also to increased RIS activity. The over-activation of RIS was best seen during the phase of continuous tapping but was also observed during the first tapping stimulus. To document the increased activation of RIS during first mechanical stimulation we aligned the data to the first tapping stimulus. The first tap already resulted in RIS activation (Fig. 4B,C). Thus, mechanical stimulation leads to both increased locomotion as well as activation of RIS. The increased depolarization of RIS may appear unexpected because RIS activity is typically associated with decreased mobility. This may be hypothetically explained by a role of RIS in dampening the increased mobilization caused by mechanical stimulation.

To test whether over-activation of RIS by mechanical stimulation is specific to the first half of sleep we started the deprivation protocol 30 minutes after lethargus onset. Again, a strong activation of the RIS neuron was seen upon sleep deprivation, albeit not as strongly as for early sleep deprivation (Fig. 4D–F). To test how RIS responds to mechanical stimulation outside of lethargus, we quantified RIS calcium activity in control animals that were stimulated outside of lethargus. While RIS also responded to tapping outside of lethargus, this response was not nearly as strong as the response during lethargus. While the RIS response was small in the L1 larvae outside of lethargus it was stronger in L2 larvae outside of lethargus, indicating that RIS activity generally increases in the course of development. In summary, RIS appears to generally respond to stimulation by increasing RIS activity, with RIS activation being much stronger during lethargus. This is in contrast to the reduced activity of mechano-sensory neurons during this stage^18^.

## Discussion

Sleep across species, including *C. elegans*, consists of different phases including sleep bouts and motion bouts^15^. To understand the complex architecture of sleep it is advantageous to do state-specific experiments. Until present, stimulation protocols are run at defined time intervals and a classification of state is performed *post hoc*^15,33,37^. Here we describe a method that allows performing experiments using real time observation of *C. elegans* behavior and detection of sleep, coupled to the execution of stimulus delivery protocols and with the capacity of following nervous system activity using calcium imaging. The major advantage of this system is that experiments do not need to be classified *post hoc* but can be precisely timed. To show the utility of this tool, we deprived worms of sleep starting with the first sleep bouts at the onset of lethargus. At present the throughput of this system is rather low, as only one animal at a time can be used for sleep deprivation. A future challenge will be to scale up the method so that multiple animals can be processed in parallel.

Mechanical stimulation is a standard procedure used to deprive animals from sleep. Sleep deprivation triggers over activation of sleep-active neurons in rodents and is believed to present increased sleep drive. By combining sleep deprivation with calcium imaging we found that the role of mechanical stimulation in activating RIS is more general than suggested by a previous study^21^. It appears that mechano-sensory systems are general activators of RIS during and outside of lethargus. However, RIS activation by mechanical stimulation is much stronger during lethargus, indicating that the nervous system is in a state that facilitates RIS activation. The observation that RIS also activates upon mechanical stimulation outside of lethargus suggests that RIS is a general part of the mechano-sensory system and also may play a role outside of lethargus. Optogenetic RIS activation also causes behavioral quiescence outside of lethargus^21,25,40^. This suggests that one functions of RIS during wakefulness is to dampen the mobilization triggered by mechanical stimulation. To some extent RIS activity can be described to be similar to a mechano-sensory neuron that typically activates upon stimulation. It has recently been shown that the ALA neuron, which induces quiescence after severe cellular stress^41,42^, also acts as a mechano-sensor^43^. In the case of the RIS neuron, it seems more likely that RIS is activated indirectly though mechano-sensory neurons, because the effects of mechanical sleep deprivation have been shown to depend on mechano-sensory neurons^32^. Also, RIS is different from mechano-sensory neurons in that it is more active during lethargus, whereas mechano-sensory neurons are less active during lethargus^18^. How precisely mechanical stimulation leads to RIS activation needs future study.

The idea of homeostasis in sleep is that sleep deprivation increases sleep pressure and thus increased sleep activation. The most likely explanation at this point is that RIS activity reflects both, homeostasis during sleep as well as response to stimulation. In sleep homeostasis, the increased activation upon stimulation of RIS is consistent with work from rodent models and previous results in *C. elegans*^21,30,31,44,45^. Our RIS data during mechanical sleep deprivation highlight the importance of considering the effects of stimulation on RIS activation when interpreting sleep deprivation data that were obtained by mechanical sleep deprivation.

## Methods

### *C. elegans* maintenance

*C. elegans* was maintained on nematode growth medium (NGM) plates seeded with *E. coli* OP50 as previously described [52]. HBR543: *unc-119(ed3) III, goeIs118*[*aptf-1-5′utr:: SL1-GCaMP3.35-SL2::mKate2-aptf-1-3′utr,unc-119*(+)]. Worms were used that were grown at temperatures between 15 °C and 25 °C. Experiments were conducted at 21.5 °C.

### Culture inside Microfluidic devices

For imaging, *C. elegans* was cultured inside agarose hydrogel microcompartments as described^35,36^. Briefly, eggs were placed together with bacterial food (*E. coli* OP50) into agarose microcompartments, 190 × 190 μm (xy dimension), 10–15 μm deep, which were cast using a PDMS mold.

### Differential interference contrast (DIC) and bright field imaging

For behavioral analysis worms were imaged inside agarose microcompartments using DIC or bright field. A Nikon Ti E inverted microscope equipped with two automated filter wheels was used. A 20 × CFI Planapochromat VC objective or a 40 × CFI S Fluor oil objective were used (both Nikon). As transmitted light source a 100 W Halogen lamp (Nikon) was used that was filtered through an infrared filter (Semrock HC785/62, round with 45 mm diameter) resulting in infrared light illumination. A standard DIC setup was used except that the analyzer was mounted into the bottom position of the filter cube of the lower filter wheel that also contained the fluorescence emission filter. An Andor Luca camera was mounted to the side port of the microscope for DIC imaging. To be able to fit four microchambers containing a single worm each onto the camera chip of the DIC camera, a 0.45 × magnification lens was introduced into the optical path of the Andor Luca camera. A custom-made aluminum adapter was made to adjust the focal plane of this camera to the focal plane of the second camera of the back port. For this, an adapter was made that consisted of two tubes inserted into each other with the camera fixed to one of the tubes and the microscope fixed to the other tube. The free ends of the tubes were inserted into each other so that the length of the tube could be adjusted. This setup resulted in a spatial resolution of approximately 1 μm/pixel. The typical frame rate for sleep detection was 1000 ms, which was set to be the default value in our sleep detection system (with a useful range being 500–4000 ms). The focal plane was adjusted empirically to match the focal plane of the iXon camera for fluorescence imaging and then this setting was fixed using screws. The Andor Luca camera was controlled by Labview. Fluorescence imaging was performed with a LED system (CoolLED) allowing GFP excitation at around 470 nm through the top filter wheel layer. LED light was passed through a GFP excitation filter (Chroma ET470/40x) and was reflected into the sample using a dicroic mirror (Chroma T495lpxr), while the emission filter slot of the filter cube was left empty. Into the lower filter wheel layer we placed a filter cube containing a dicroic mirror reflecting fluorescence light (Semrock HC705lp) through a GFP emission filter (Chroma ET525/50 m) onto a second camera for calcium imaging. The bottom filter slot of this filter cube contained the DIC analyzer. For calcium imaging we used an Andor iXon DU897 EMCCD camera using short exposure times of 5 ms to avoid blurring. The LED was on only during the short camera exposure time using the TTL signal from the iXon “fire” output. The iXon camera was controlled by Andor iQ2 software to take time-lapse movies with a 4 s interval. The EM gain of the camera was set between 100 and 250. LED intensity was in the range of 1 mW/mm^2^ to 5 mW/mm^2^. This level of blue-light illumination did not cause detectable bleaching or any noticeable behavioral changes.

### Automated sleep detection setup

Sleep detection is based on custom written LABVIEW routines (described in detail in supplement (supplemental file describing the “*C. elegans* automated sleep deprivation” program) and in a PhD thesis written by Jan Spies available online (http://hdl.handle.net/11858/00-1735-0000-0028-867B-8)). All LABVIEW files can be found and downloaded from GitHub (https://github.com/HenrikHBR/sleepdeprive-Labview). Sleep deprivation is triggered by LABVIEW using TTL outputs of the National Instruments PCIe-6509 data acquisition card. The data acquisition card is connected by a custom-made adapter and BNC cables to the external TTL trigger input of the deprivation device, a mechanical tapper similar to described previously^18^, except that a Kuhnke H2286 magnet was used. Briefly, the dish-tapper used consisted of a custom-made aluminum holder for a sample and a piston that gets accelerated by an electromagnet as described. The magnet holder ensured that the tapping stimulus gets delivered horizontally in the plane of the sample, restricting sample movement mainly to the xy-plane. Image acquisition and dish tapping were shuttered using TTL signals in such a way that no apparent blurring due to tapping-induced movement occurred. The standard tapping stimulus for sleep deprivation experiments consisted of 5 taps that were administered in a 1 s interval.

### Detection of sleep by frame subtraction

The image subtraction value at the time point t for the pixel (i, j) is equal to the absolute difference of the pixel (i, j) value for the time points t and t + 1 (equation 1).

1 $$ImSub(t{)}_{ij}=\parallel Im(t+1{)}_{ij}-Im(t{)}_{ij}\parallel $$

With t: Point in time, i, j: Indices of pixel values.

By summing up the image subtraction values of all pixels one obtains the image subtraction value of the entire picture (equation 2).

2 $$Imsub(t)={\rm{\Sigma }}ijImSub(t{)}_{i,j}$$

To quantify how well image subtraction is reflecting mobility we compared manually tracked nose velocities to their corresponding image subtraction values. Image subtraction closely reflected nose velocity. The wake-sleep-wake time course of image subtraction and nose velocity showed a similar pattern. Both showed a dip that corresponds closely to the sleep period. The Spearman rank test gave a positive correlation between image subtraction and nose velocity. The corresponding Spearman correlation coefficient was 0.595.

Thus, image subtraction is a measure for the mobility of the worm. Does image subtraction permit robust recognition of the sleep state of the worm? Recognition of the sleep state is based on the low mobility of the worm and the resulting low image subtraction values. The questions to answer are therefore: First, is low immobility a robust indicator for the sleep state. Second, is the method of image subtraction suited to detect this immobility during sleep? The sleep detection criterion should be applicable to a wide range of sleep mutants, varying illumination settings and magnifications. Therefore it was decided not to use an absolute threshold, but a relative threshold, which is dependent on prior mobility. We established the sleep criterion empirically based on a dataset of the wake sleep cycle of ten animals. We annotated sleep and wake timings and tested which threshold best detected the sleep onset and did not result in false positives during wake state. We decided to use two separate thresholds. For a local time window to be defined, the standard deviation has to be smaller than a fraction of the global standard deviation (equation 3) and the mean value has to be smaller than a fraction of the global mean value minus the difference between the global mean and the global minimum (equation 4).

3 $$std(local)\le std(global)/{k}_{std}$$

4 $$mean(local)\le mean(global)-(mean(global)-min(global))/{k}_{mean}$$

The second equation can be rewritten as follows (equation 5).

5 $$mean(local)\le (({k}_{mean}-1)\ast mean(global)+min(global))/{k}_{mean}$$

With *kstd*, *kmean*: empirically determined constants that depend on the local time window observed. For wild-type worms a time window of five frames, *k*1 = 40 and *k*2 = 1.5 yielded robust detection of the sleep state. Using these parameters the above equations 3 and 4 can be rewritten (equations 6 and 7).

6 $$std(Imsub({t}_{n-4,\ldots ,n}))\le std(Imsub({t}_{0,\ldots ,n}))/40$$

7 $$mean(Imsub({t}_{n-4,\ldots ,n}))\le (0.5\ast mean(Imsub({t}_{0,\ldots ,n}))+min(Imsub({t}_{0,\ldots ,n})))/1.5$$

With *t*0: time point of first image subtraction value, *tn*: last point in time we obtain the following equation (equation 8).

8 $$Imsub(t)={\rm{\Sigma }}\,ijImSub(t{)}_{i,j}$$

We evaluated the sleep detection criterion for 25 animals that were not implicated in the postulation of the sleep detection criterion. In 89.3% of the cases sleep was detected during the non-pumping lethargus period and allowing a tolerance of 5 min prior to non-pumping start 96.4% was detected correctly. Sleep was detected on average with a delay of 5.1 minutes after lethargus start. We implemented the sleep detection criterion as part of the “C. elegans automated sleep deprivation” program in LABVIEW. See Supplemental Information for details on the operation of the program.

## Electronic supplementary material

Supplementary Information
