# Supplementary material for: Automated detection and manipulation of sleep in C. elegans reveals depolarization of a sleep-active neuron during mechanical stimulation-induced sleep deprivation
Source: Sci Rep. 2018 Jun 27;8:9732. doi: 10.1038/s41598-018-28095-5 (PMC6021397; doi:10.1038/s41598-018-28095-5)
Supplement: Supplementary file 1 — Supplementary Information [file 41598_2018_28095_MOESM1_ESM.pdf]

Supplementary information for

Automated detection and manipulation of sleep in *C. elegans* reveals depolarization of a sleep-active neuron during mechanical stimulation-induced sleep deprivation

Authors: Jan Spies, Henrik Bringmann*

Annotation of the Labview program: "*C. elegans* automated sleep deprivation"

The "*C. elegans* automated sleep deprivation" program was written in LABVIEW. LABVIEW was chosen for the following reasons:

- A pre-existing integration of interfaces for many devices, like cameras and data acquisition cards

- A large library containing functions for data acquisition, mathematics, statistics, and most important an advanced graphical user interface (GUI)

- A graphical programming approach, that makes it easy for future users not having a background in programming to modify the program accordingly to their needs

LabVIEW functions/routines are called virtual instruments (VIs). Instead of a written source code each VI consists of a block diagram, a front panel and a connector panel. The block diagram is the graphical equivalent of the written source code. The front panel is a graphical user interface corresponding to the block diagram that is automatically created by LABVIEW. The connector panel represents the input and output parameters of the VI that have to be used by another VI to call this VI. In the following paragraph we explain the source code of the LABVIEW "*C. elegans* automated sleep deprivation" program. The "*C. elegans* automated sleep deprivation" program is based on the call of 9 subroutines that are visualized in the scheme below (Figure 1).

Program scheme

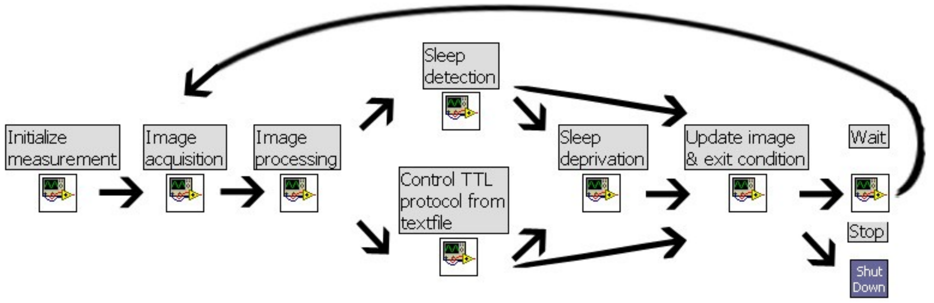

Figure 1: Program scheme

A measurement always starts with the "Initialize measurement VI", that is followed by the "Image acquisition VI" and the "Image processing VI". Depending on whether sleep deprivation or control mode is chosen, either the "Sleep detection VI" or the "Control TTL protocol from textfile VI" is executed. In case of a successful sleep detection "Sleep deprivation VI" follows, or in case of no sleep detection the program continues directly with "Update image detection & exit condition VI". Evaluation of the exit condition either runs the "Wait VI" or shuts down the measurement with the "ShutDown VI". The "Wait VI" then triggers again the "Image acquisition VI" leading to a cyclic VI execution.

The LABVIEW block diagram implementation of this scheme is shown in Figure 2. Additionally to the scheme, a mode prior start measurement has been added to setup the image. If in the mode prior start measurement the program loops after the "Image acquisition VI" directly to the "Wait VI”.

Main

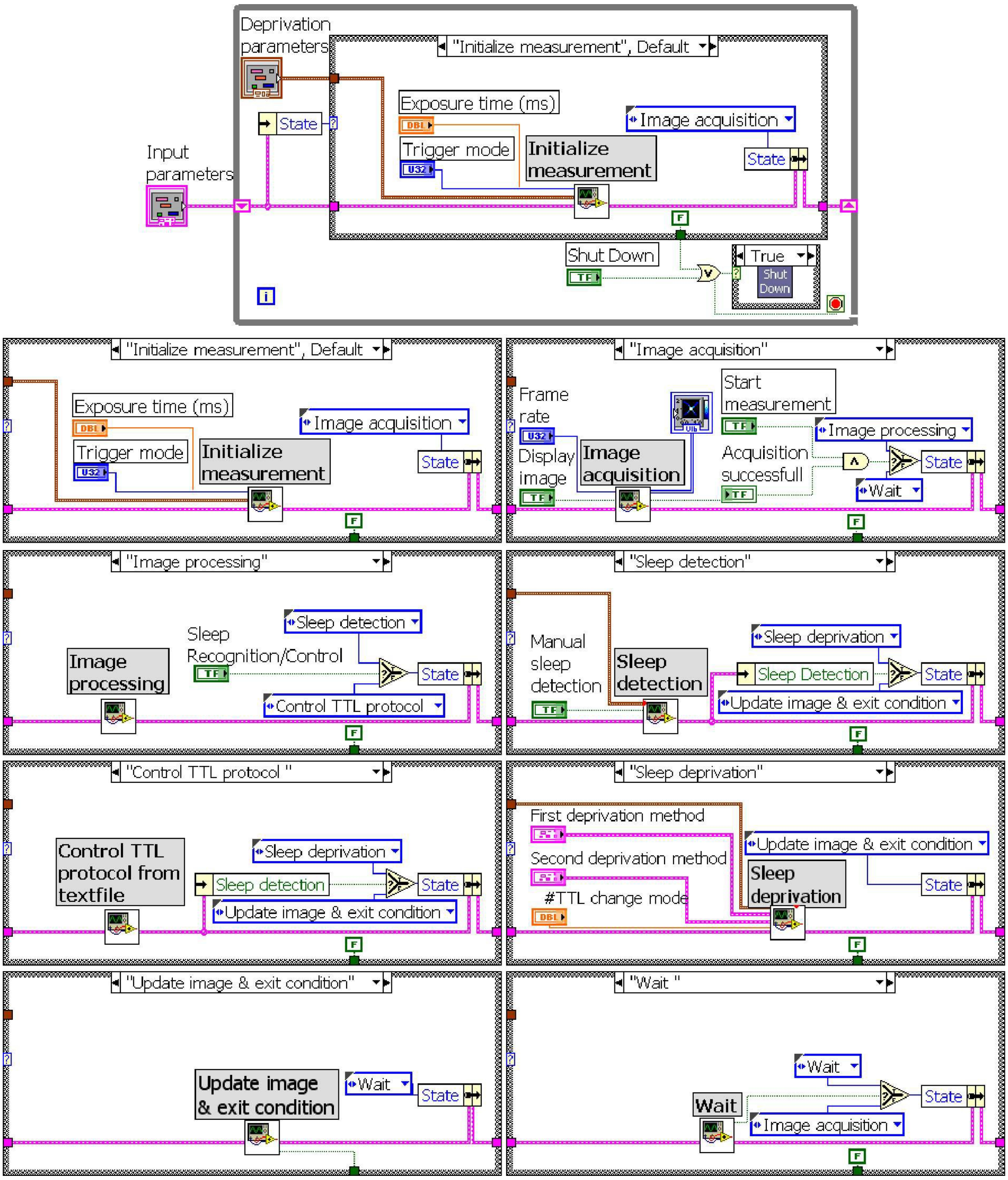

Figure 2: Maind1ALLIncl

Initialize measurement

The "Initialize measurement VI" (Figure 3) sets up the parameters used during the measurement ("Global and local image subtraction arrays VI", "Multiple animals parameters VI", "Single animal subimage pixels VI"), creates the folders to save the

measurement files ("Create folder VI") and initializes the camera settings ("Initialize camera settings VI"). The "Initialize measurement VI" is run only once at the beginning of a measurement.

Initialize measurement

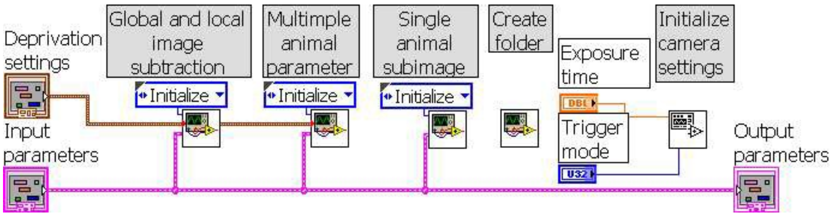

Figure 3: Initialize measurement

The "Global and local image subtraction array VI" (Figure 4) stores the global and local image subtraction values that are used to evaluate the sleep detection criterion. At this time point the "Global and local image subtraction array VI" is initialized. Setting up a one-dimensional array of length "# Images for sleep detection" filled with zeros for the local image subtraction array. And a one-dimensional array with one zero element for the global image subtraction array.

Global and local image subtraction array

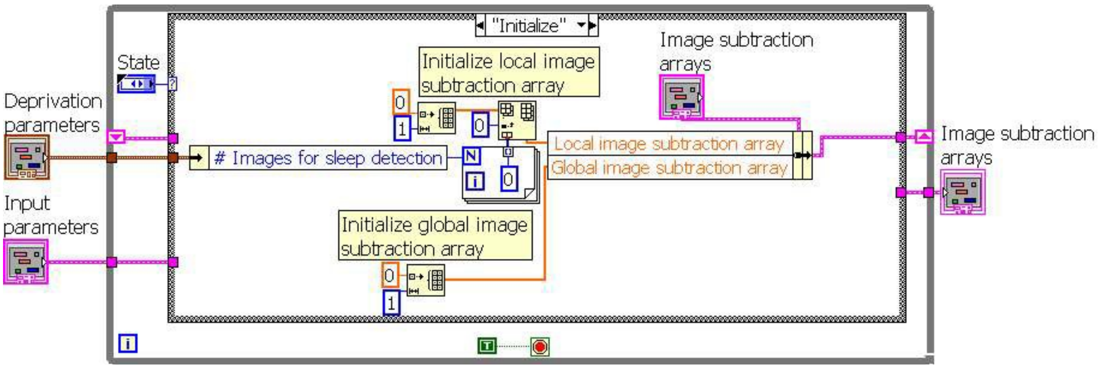

Figure 4: Global and local image subtraction array

The "Multiple animal parameters VI" (Figure 5) calculates the index of the sleep deprivation animal and the total number of animals.

Multiple animal parameters

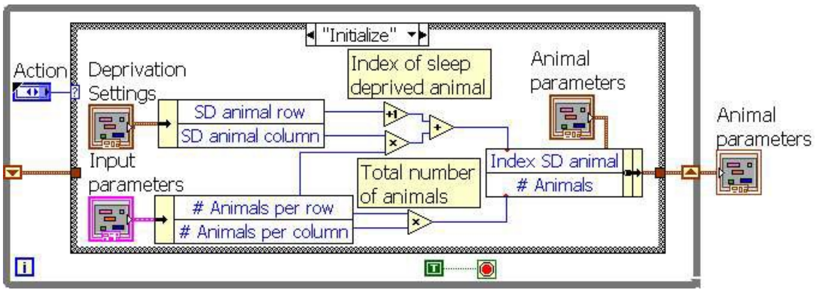

Figure 5: Multiple animal parameters

The "Single animal subimage pixel ranges VI" (Figure 6) calculates from the total number of pixels of the camera, the pixel ranges corresponding to single animals. It does so by dividing the total image into equally sized subimages according to the # animals per row/column.

Single animal subimage pixel ranges

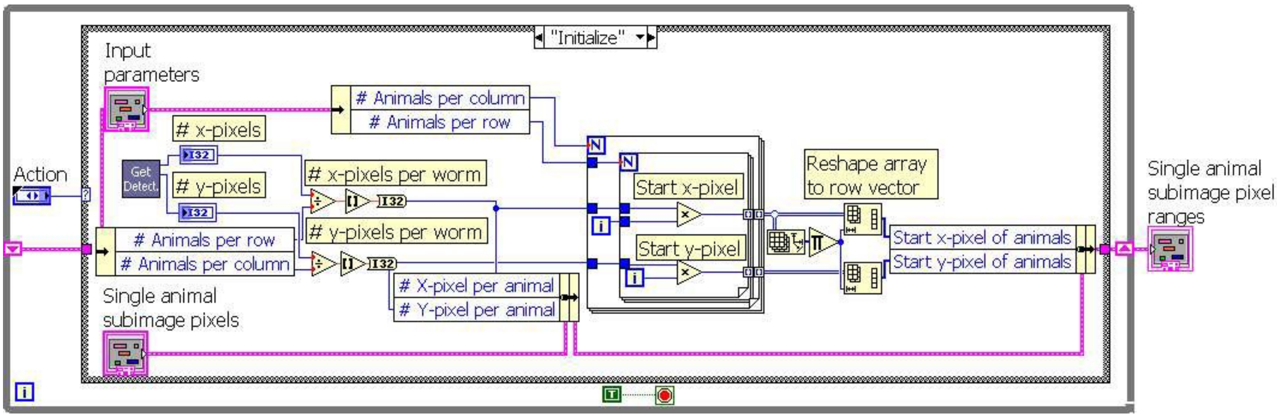

Figure 6: Single animal subimage pixel ranges

The "Create folder VI" (Figure 7) verifies whether a folder with the same strain name and date already exists. If no folder exists it creates a new folder. If the folder already exists it gives an error message. In this case the user should rename or copy the pre- existing folder to another location. If the user continues the measurement despite an error message the old measurement data is overwritten.

Create folder

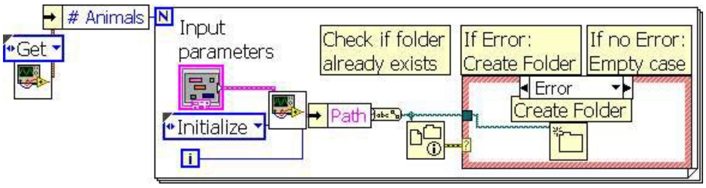

Figure 7: Create folder

The "Initialize camera settings VI" (Figure 8) starts the initialization of the camera and sets up the camera parameters used for the measurement. Of the parameters set here only the acquisition time and trigger mode should be varied using the main front panel.

Initialize camera settings

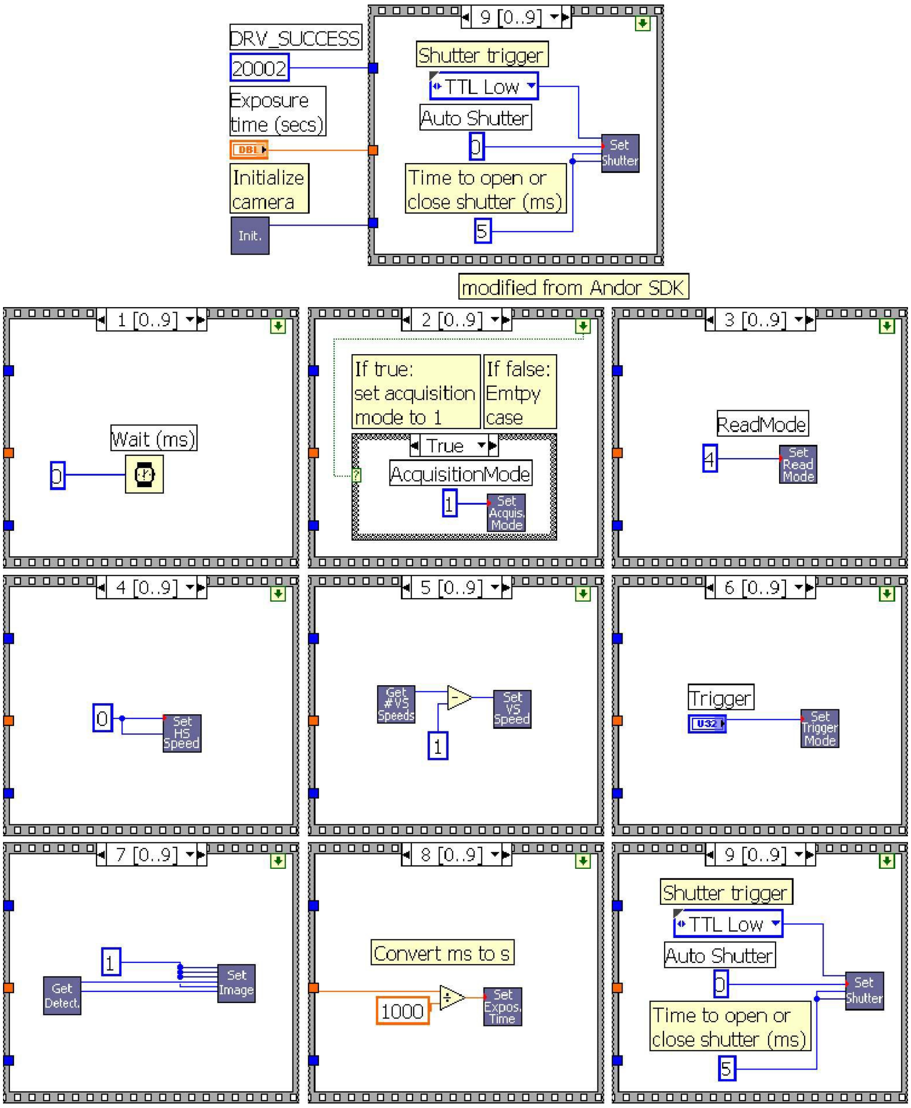

Figure 8: Initialize camera settings

Image acquisition

The "Image acquisition VI" (Figure 9) starts the image acquisition ("Start

acquisition VI") and if the acquisition is successful it waits for the end of acquisition ("Wait for end of acquisition VI") and stores a converted version of it for display and further image processing ("Get image VI").

Image acquisition

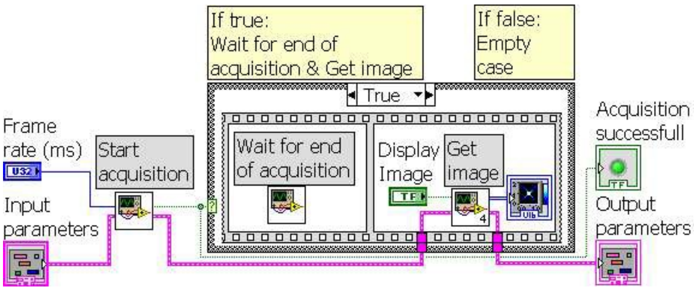

Figure 9: Image acquisition

The "Start acquisition VI" (Figure 10) adjusts the frame rate to account for delays due to the program execution, tries to start the acquisition and outputs the acquisition time and whether the acquisition was successfully started.

Start acquisition

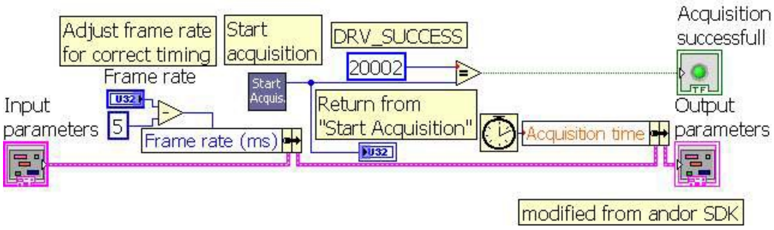

Figure 10: Start acquisition

The "Wait for end of acquisition VI" (Figure 11) verifies if the image acquisition has been completed. As long as this is not the case it introduces a 5 ms delay and then repeats the verification.

Wait for end of acquisition

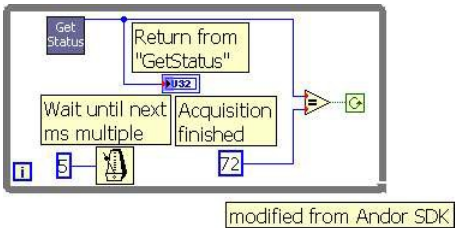

Figure 11: Wait for end of acquisition VI

The "Get image VI" (Figure 12) converts the acquired image that is stored in a one-dimensional array to a two-dimensional array, corresponding to the image dimensions. This converted image is stored for further image processing. If "display image" is chosen in the main front panel the image is displayed.

Get image

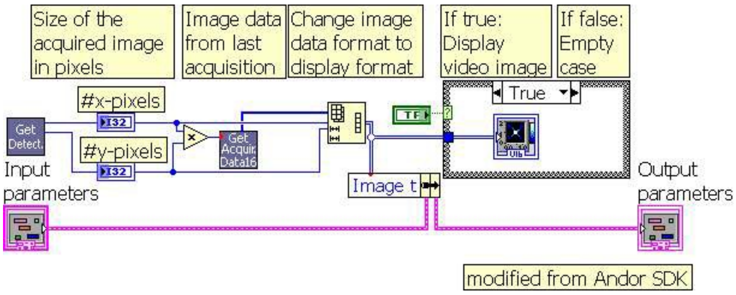

Figure 12: Get image

Image processing

The "Image processing VI" (Figure 13) saves single animal image subselections, calculates and saves corresponding image subtraction values and determines the image subtraction value of the sleep deprivation animal. For each animal it generates new file- and pathnames ("File-pathname VI"), creates the image subselection ("Create image subselection VI"), saves it as a tiff file ("Save array to image VI") and saves the image acquisition time to a textfile ("Save image acquisition time to textfile VI"). If images have been acquired before it calculates the image subselection of the previous image as well ("Create image subselection VI"), then calculates the image subtraction value ("Calculate image subtraction value VI"), saves it to a text file ("Save image subtraction value VI") and determines and outputs the image subtraction value of the sleep deprivation animal ("Select image subtraction value of SD animal VI").

Image processing

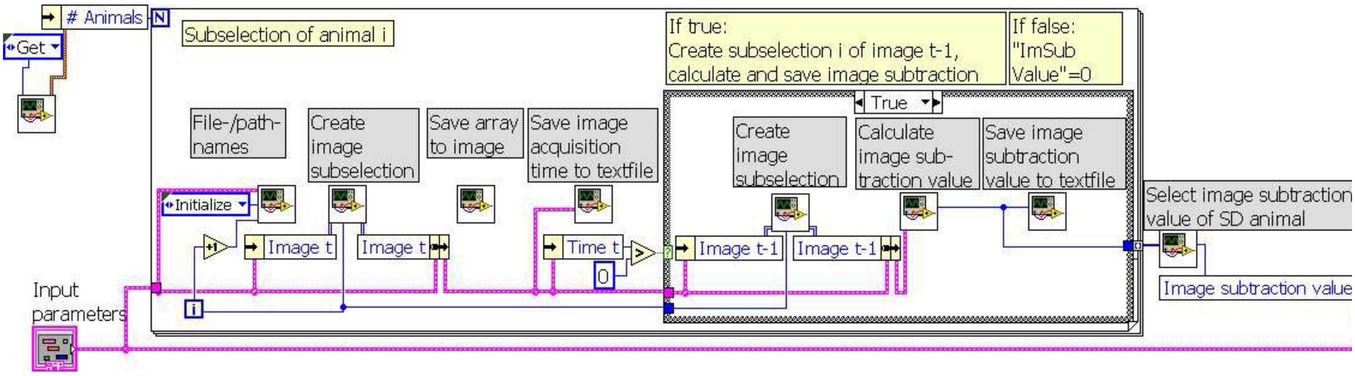

Figure 13: Image processing

The "File-pathname VI" (Figure 14) generates and outputs new file- and pathnames for the animal with the index i. Calling it with the initialization option initializes the path, the filestem, and the filestem including the current time t and outputs them. The filestem denominates the filename preceded by the path, but without file extension.

File-pathname

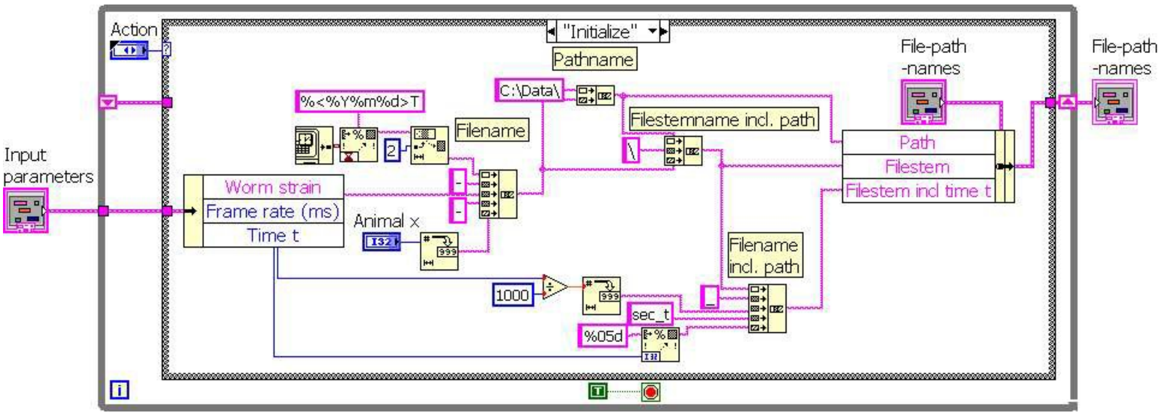

Figure 14: File-pathname

The "Create image subselection VI" (Figure 15) determines and outputs the image subselection of the full image that corresponds to the single animal with index i.

Create image subselection

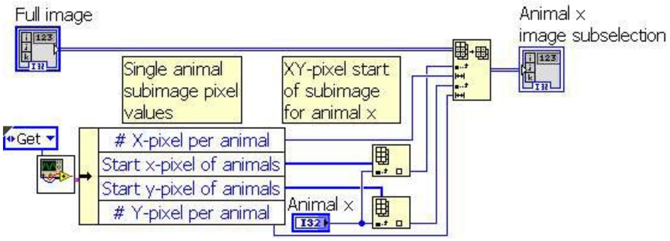

Figure 15: Create image subselection

The "Save array to image VI" (Figure 16) saves the subselection image array as a 16 bit grayscale tiff file. To output the image array as a tiff file the following routines that are included in the LABVIEW IMAQ Vision are used: "IMAQ Create", "IMAQ ArrayToImage", "IMAQ GetPalette" and "IMAQ WriteFile".

Save array to image

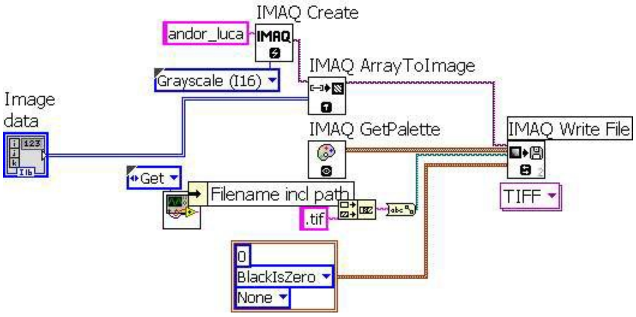

Figure 16: Save array to image

The "Save image acquisition time to text file VI" (Figure 17) saves the image acquisition time to the text file named "Strain-Name-AnimalIndex-Date-time-loc.txt".

Save image acquisition time to text file

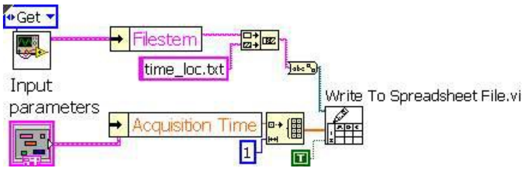

Figure 17: Save image acquisition time to text file

The "Calculate image subtraction value VI" (Figure 18) calculates the image subtraction value, outputs and stores it.

Calculate image subtraction value

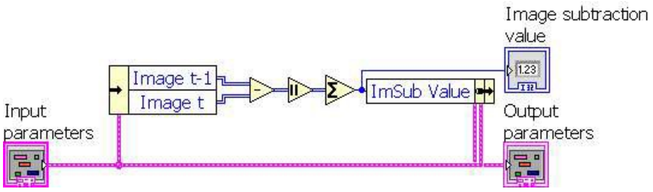

Figure 18: Calculate image subtraction value

The "Save image subtraction value 18" (Figure 19) saves the image subtraction value to the text file named "Strain-Name-AnimalIndex-Date-subtraction.txt"

Save image subtraction value

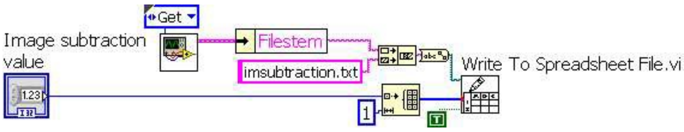

Figure 19: Save image subtraction value

The "Select image subtraction value of SD animal VI" (Figure 19) determines the image subtraction value of the sleep deprivation animal and outputs it.

Select image subtraction value of SD animal

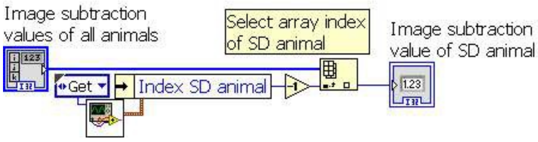

Figure 19: Select image subtraction value of SD animal

Sleep detection

The "Sleep detection VI" (Figure 20) combines a verification of the sleep detection prerequisites, an automated and/or manual sleep detection, and a user defined optional delay of sleep detection. Local and global image subtraction arrays get updated ("Global and Local image subtraction arrays VI") and prerequisites to start sleep detection are evaluated ("Prerequisites to start sleep detection VI"). If the prerequisites are not fulfilled the VI terminates with the output "sleep detection"="false". If the prerequisites of sleep detection are fulfilled automated sleep detection criteria are evaluated ("Automated sleep detection mode VI"), manual sleep detection is evaluated ("Manual sleep detection VI") and a user defined optional delay for the sleep deprivation start is evaluated ("Delay sleep deprivation start VI").

Sleep detection

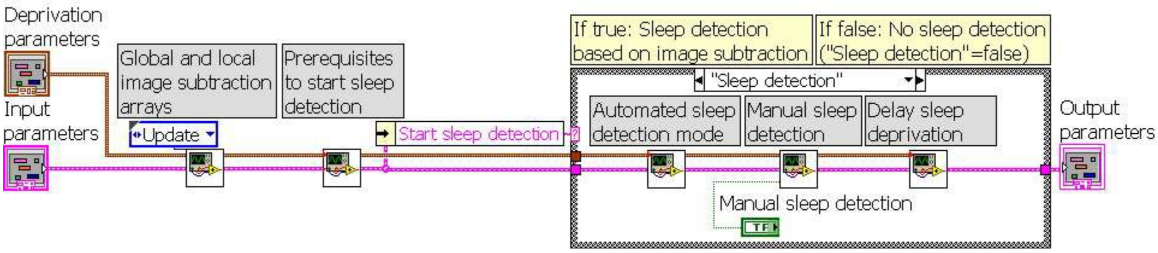

Figure 20: Sleep detection

The "Global and local image subtraction array VI" (Figure 21) called with the update argument ads the latest image subtraction value of the SD animal to the global and local image subtraction arrays. During the update process the local image subtraction array is reduced by the oldest image sub- traction value and the latest image subtraction value is added to it. The global image subtraction array gets extended by the latest image subtraction value.

Global and local image subtraction arrays

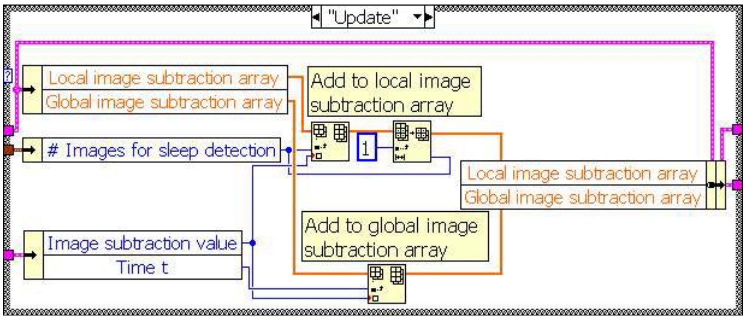

Figure 21: Global and local image subtraction arrays

The "Prerequisites to start sleep detection VI" (Figure 22) determines whether different prerequisite criteria are fulfilled to start sleep detection. It verifies that the start time for sleep detection is reached, that the minimum interval between TTL pulses is kept, and that neither the maximum number of adjacent TTL, nor the maximum deprivation time, nor the maximum number of TTL is reached.

Prerequisites to start sleep detection

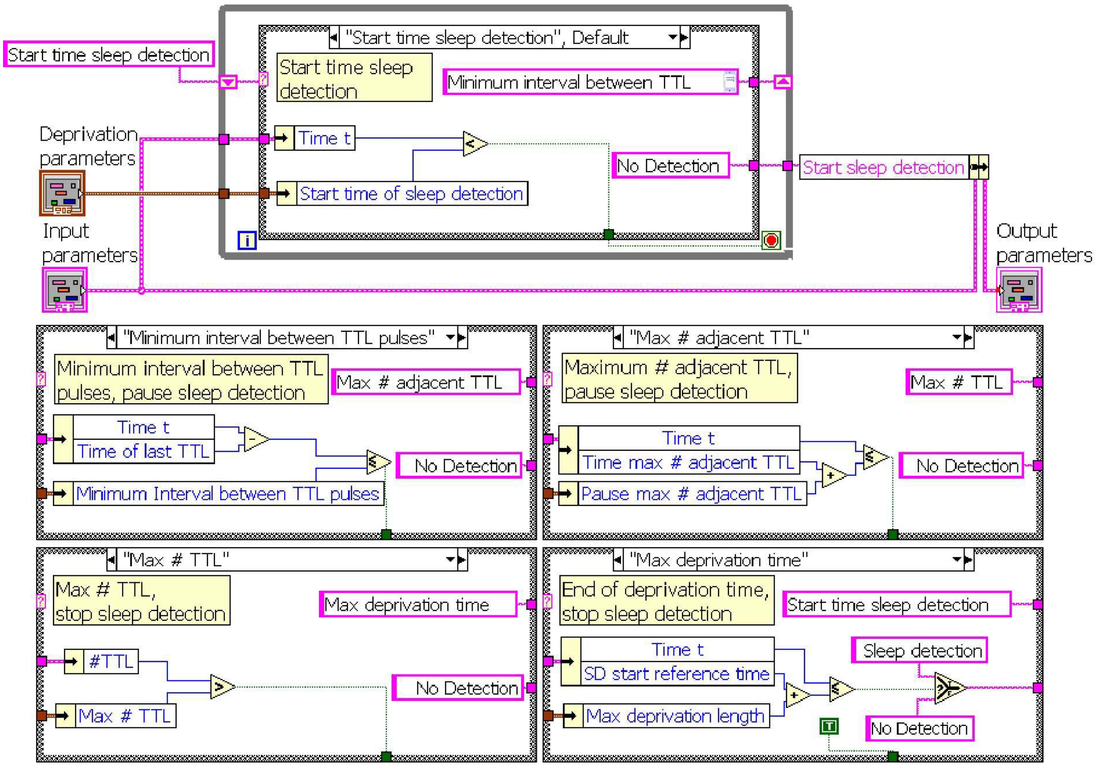

Figure 22: Prerequisites to start sleep detection

The "Automated sleep detection mode VI" (Figure 23) determines which sleep detection criterion to employ. Total immobility ("Sleep detection criterion total immobility VI") is used as the sleep detection criterion for a user-defined number of total immobility detections as reflected by the number of TTL signals ("#TTL"). Once sleep has been detected and deprived more often a less restrictive sleep criterion is used ("Sleep detection criterion low mobility VI").

Automated sleep detection mode

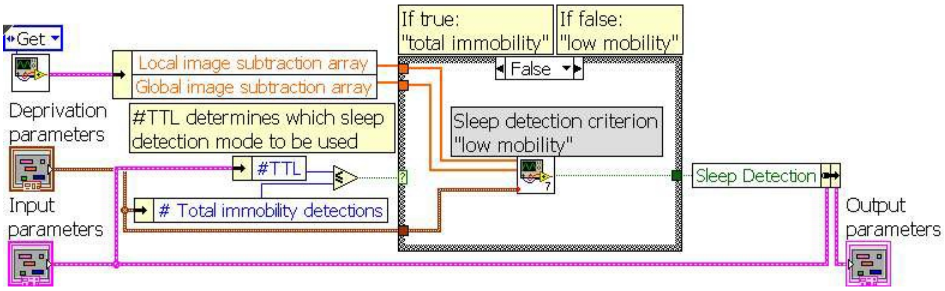

Figure 23: Automated sleep detection mode

The "Sleep detection criterion total immobility VI" (Figure 24) evaluates the empirical sleep detection criterion based on image subtraction values.

Sleep detection criterion total immobility

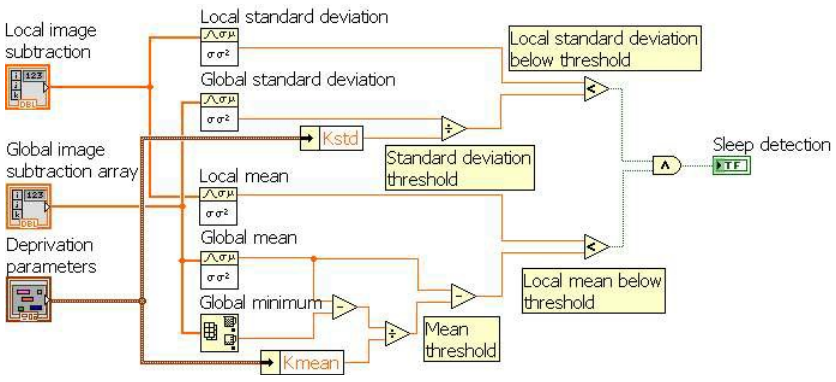

Figure 24: Sleep detection criterion total immobility

The "Sleep detection criterion low mobility VI" (Figure 25) is detecting sleep if the mobility for only one time point is lower than a threshold.

Sleep detection criterion low mobility

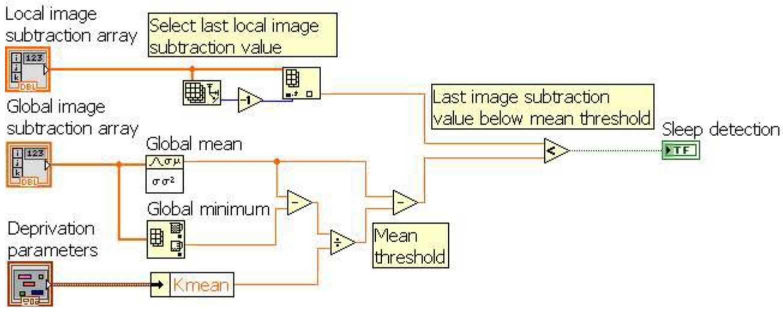

Figure 25: Sleep detection criterion low mobility

The "Manual sleep detection VI" (Figure 26) determines, whether sleep has been detected manually by pressing the button "manual sleep detection" in the main front panel. The VI outputs "sleep detection"="true" if sleep has been either detected automatically and/or manually.

Manual sleep detection

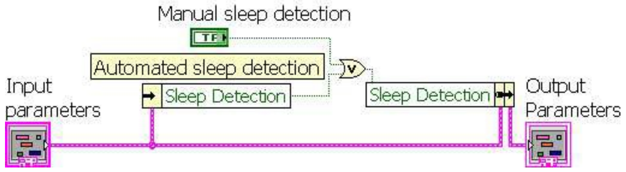

Figure 26: Manual sleep detection

The "Delay sleep deprivation start VI" (Figure 27) verifies if a sleep detection gets delayed by user defined optional parameters. First sleep detection parameters are up- dated ("Update sleep detection parameters VI"), then it is verified whether the start of sleep deprivation gets delayed by a certain amount of images ("Delay deprivation start by x images VI") or of sleep detections ("Delay deprivation start by x detections").

Delay sleep deprivation start

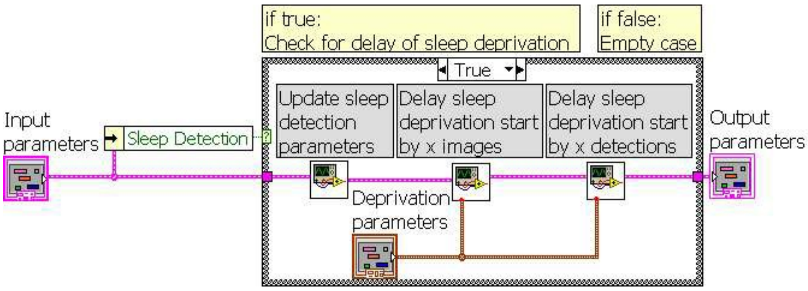

Figure 27: Delay sleep deprivation start

The "Update sleep detection parameters VI" (Figure 28) increases the number of sleep detections by 1 and if this is the first sleep detection, sets "Time of first sleep detection" to the current time.

Update sleep detection parameters

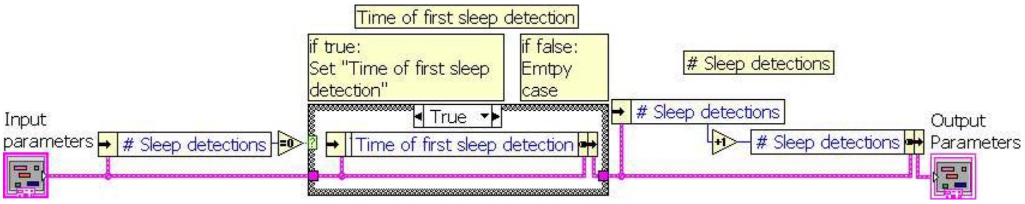

Figure 28: Update sleep detection parameters

The "Delay deprivation start by x images VI" (Figure 29) verifies if the sleep deprivation start gets delayed by a user- defined amount of time.

Delay deprivation start by x images

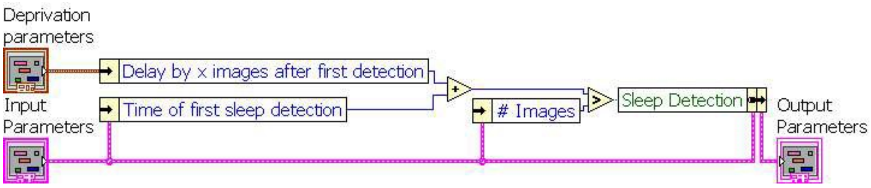

Figure 29: Delay deprivation start by x images

The "Delay deprivation start by x detections VI" (Figure 30) verifies if the sleep deprivation start gets delayed by a user- defined amount of detections.

Delay deprivation start by x detections

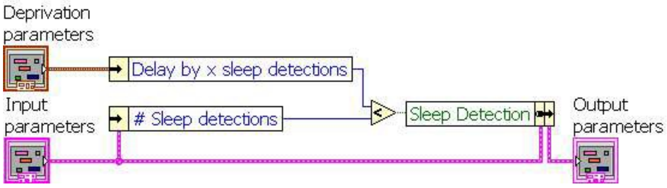

Figure 30: Delay deprivation start by x detections

Control TTL protocol

The "Control TTL protocol VI" (Figure 31) reads a list of TTL timings from a textfile, compares them to the current time t and outputs "sleep detection"="true" if they coincide.

Control TTL protocol

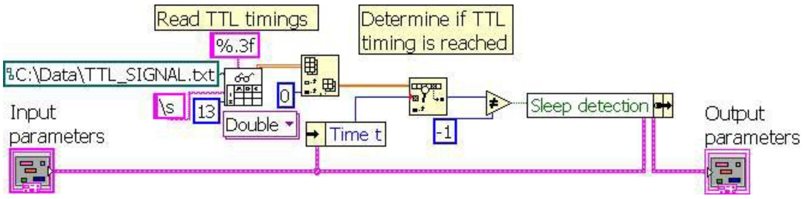

Figure 31: Control TTL protocol

Sleep deprivation

The "Sleep deprivation VI" (Figure 32) saves and updates TTL parameters, triggers optional calcium imaging, determines the deprivation method to be used and triggers the thereby specified TTL pulses.

Sleep deprivation

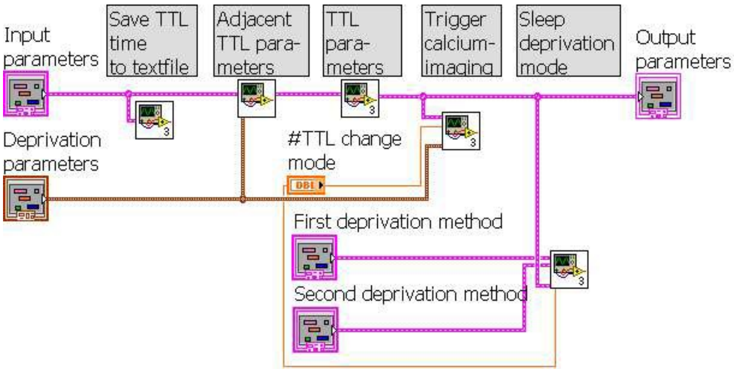

Figure 32: Sleep deprivation

The "Save TTL time to textfile VI" (Figure 33) saves the current time to the textfile "Strain-name-IndexSDAnimal-Date-TTL_SIGNAL.txt".

Save ttl time to textfile

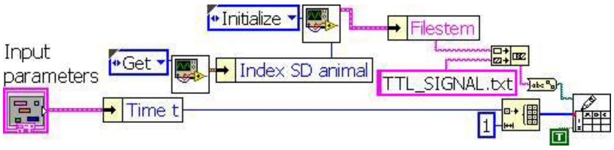

Figure 33: Save ttl time to textfile

The "Update adjacent TTL parameters VI" (Figure 34) updates the number of adjacent TTL and if the number of adjacent TTL corresponds to the user defined maximum number of adjacent TTL it sets "time maximum # adjacent TTL" to the current time.

Update adjacent TTL parameters

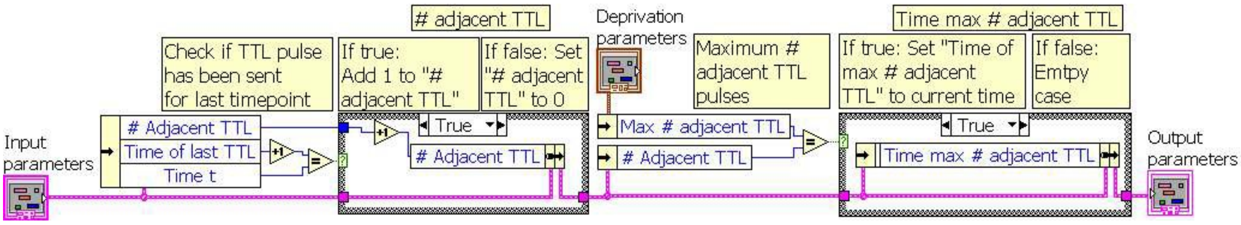

Figure 34: Update adjacent TTL parameters

The "Update TTL parameters VI" (Figure 35) updates the number of TTL, sets the time of the last TTL to the current time, and if the number of TTL corresponds to the user defined "# TTL SD start reference time" it sets "SD start reference time" to the current time.

Update TTL parameters

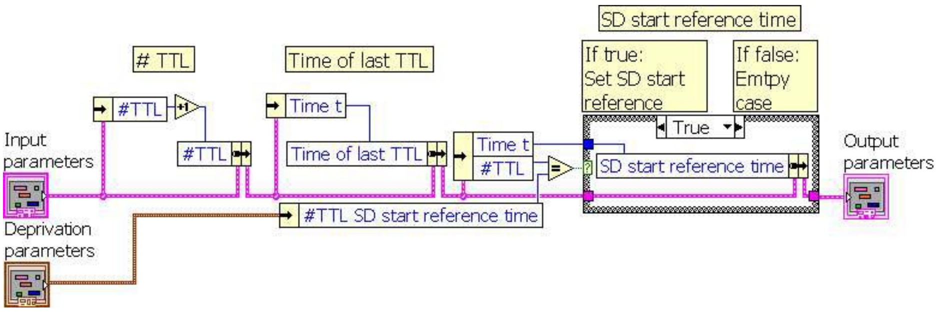

Figure 35: Update TTL parameters

The "Trigger calcium-imaging VI" (Figure 36) triggers a user defined optional calcium- imaging. Mode "0" corresponds to no calcium imaging. Mode "1" outputs a single TTL pulse to the "external trigger" of an EMCCD-camera used for calcium imaging. Mode "2" outputs a single TTL pulse to the "external trigger" of a EMCCD-camera used for calcium imaging if the number of TTL is equal or bigger than "# TTL change mode".

Trigger calcium imaging

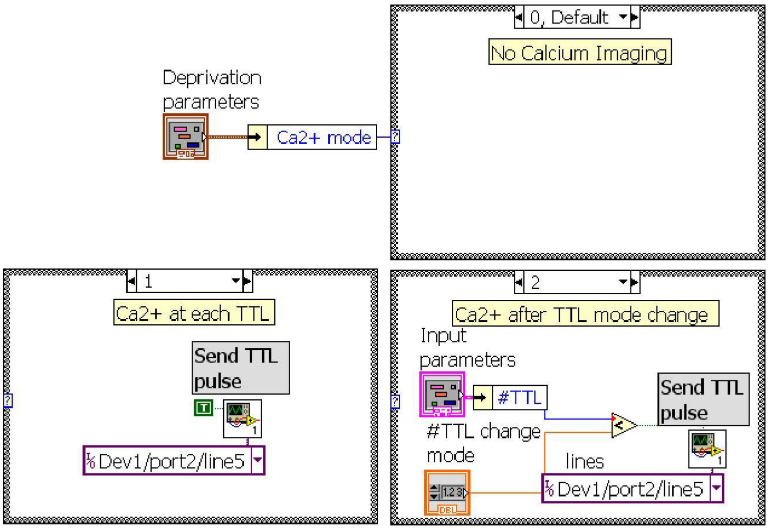

Figure 36: Trigger calcium imaging

The "Sleep deprivation mode VI" (Figure 37) determines which one out of two user-defined deprivation methods is used depending on the "# TTL" compared to "# TTL change mode". It then outputs a TTL pulse sequence with the parameters specified by the selected sleep deprivation method.

Sleep deprivation mode

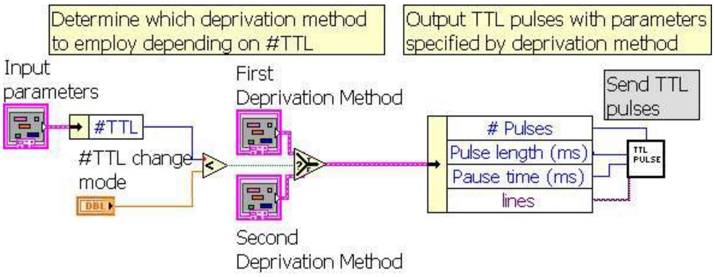

Figure 37: Sleep deprivation mode

The "Send TTL pulses VI" (Figure 38) outputs a sequence of TTL pulses defined by the user defined values of the deprivation method: "# Pulses", "Pulse length (ms)", "Pause time (ms)", and "Line to output TTL".

Send TTL pulses

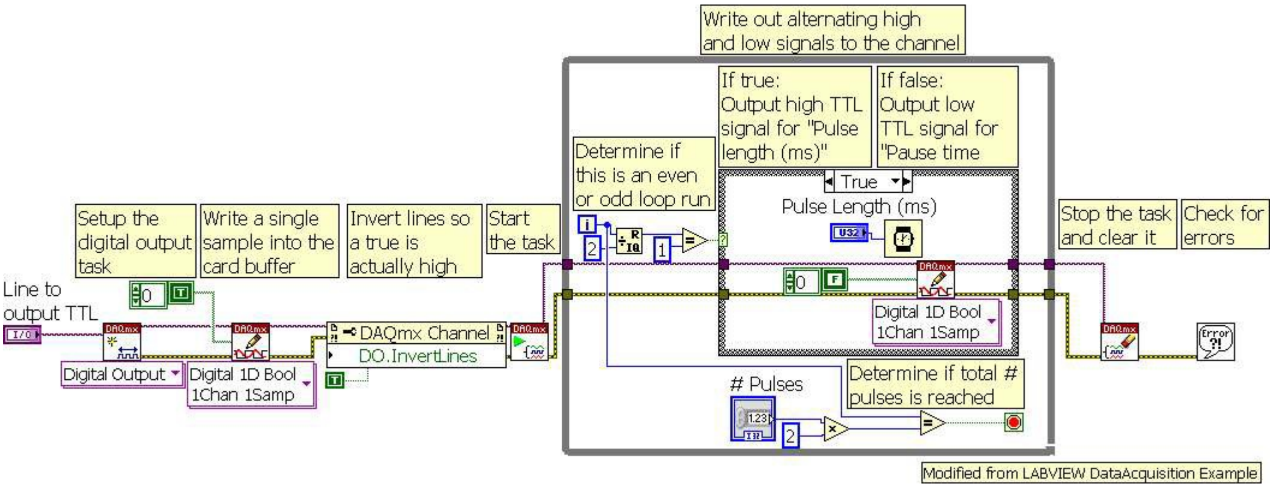

Figure 38: Send TTL pulses

Update image & exit condition

The Update image & exit condition VI replaces image t-1 by image t, increases the time t by one, and verifies whether the user defined end measurement time (Max_num_runs) is reached (Figure 39). If the end measurement time is reached, the camera gets shut down using the ANDOR SDK VI ShutDown and the Main VI terminates.

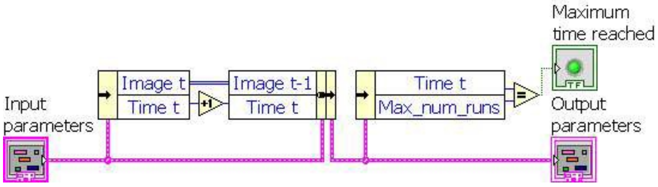

Figure 39: Update image and exit conditions.

Wait

The "Wait VI" (Figure 40) delays the next image acquisition until the user defined frame rate is reached.

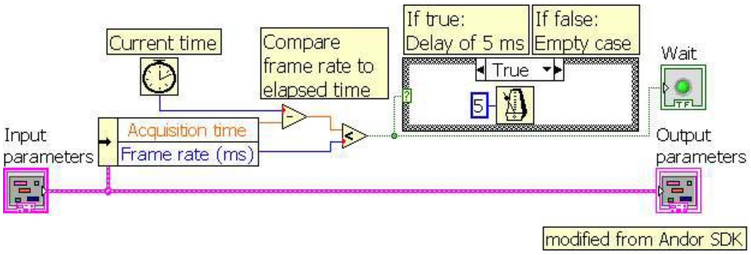

Figure 40: Wait

"*C. elegans* automated sleep deprivation" interface

"*C. elegans* automated sleep deprivation" program interface consist of a single control window (Figure 41). The controls can be divided in six major categories that are indicated in the figure by red capital letters:

- A: Display of camera image,
- B: Camera acquisition settings
- C: TTL-pulse settings
- D: General settings
- E: Sleep deprivation settings
- F: Start/End measurement

Annotation of the Labview program “*C. elegans* automated sleep deprivation”

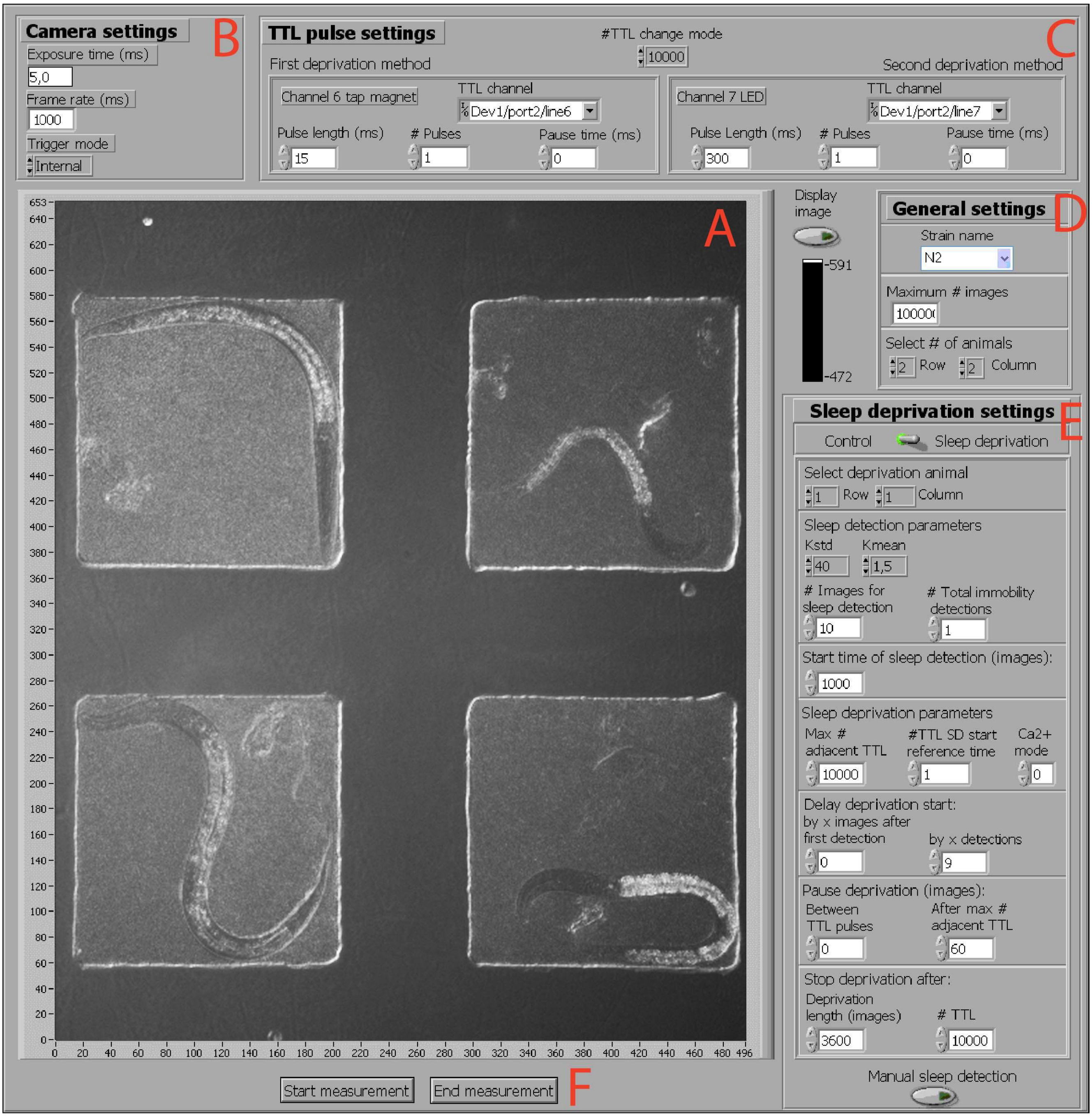

Figure 41: Control window - A: Display of camera image, B: Camera acquisition settings, C: TTL-pulse settings, D: General settings, E: Sleep deprivation settings, F: Start/End measurement

Display image

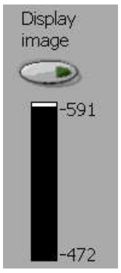

Figure 42: Display image

- Display image (Figure 42): Default (off), range (on, off).

If switched on newest image is displayed and gets updated by timing specified in "frame rate (ms)". Before starting the measurement display image should be switched off, to avoid delays in capturing images.

- Light intensity: range (20%-80% of saturation intensity).

Minimum and maximum pixel light intensity. If using an Andor Luca camera, intensities of about 7000-9000 are a good choice.

Camera settings

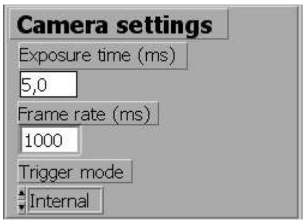

Figure 43: Camera settings

Figure 43 shows the camera settings:

- Exposure Time (ms): Default value (5), range (1-10)

- Frame rate (ms): Default value (1000), range (500-4000)

- Trigger mode: Default Internal, range (Internal, External)

Trigger mode configures whether the image acquisition of the camera should be triggered by LABVIEW (Internal), or whether the camera gets trigger by a TTL signal (External).

TTL-pulse settings

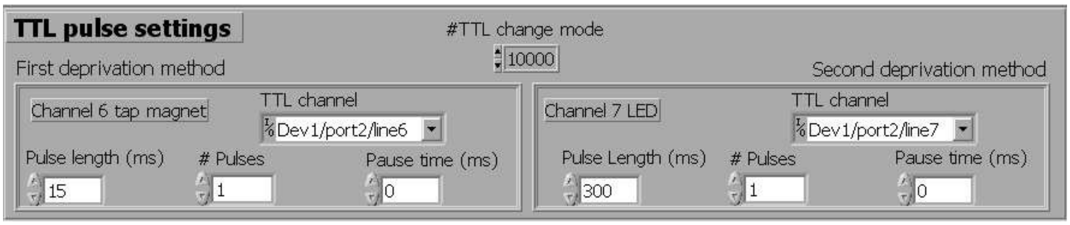

Figure 44: TTL pulse settings

Figure 44 shows the TTL pulse settings:

- # TTL change mode: Default value (10000), range (0-100000).

Number of TTL signals after which the deprivation method changes. Deprivation methods are defined by the following settings.

- First/second deprivation method:

TTL channel: Default value (Line6/Line7), range (Line1-Line8).
Channel of the data acquisition card that should output TTL signals. In our setup, currently line-6 is connected to a magnetic dish-tapper and line-7 to a LED.

Pulse length (ms): Default value (15/300), range (5-10000).
Duration of one TTL pulse in ms.

pulses: Default value (1/1), range (1-100). Number of separate TTL pulses that are output as one bout.

Pause time (ms): Default value (0/0), range(0-10000). Pause time between two TTL pulses that belong to one output signal.

General settings

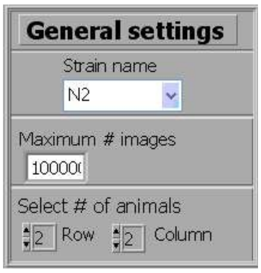

Figure 45: General settings

Figure 45 shows the general settings:

- Strain name: Default value (N2). Range (any string)

The strain name is used together with the date as the name for the measurement. New *C. elegans* strain names can be added by right click, edit item.

- Maximum # (number) images: Default value (100000), range (1000-200000).
The measurement will be stopped automatically if this value is reached.

- Select # of animals row/column: Default value (2/2), range(1-3, 1-3).
Sets the number of animals per row/column. Depending on the size of the camera chip and objective used, more than 3x3 animals can be imaged and sleep be detected.

Sleep deprivation settings

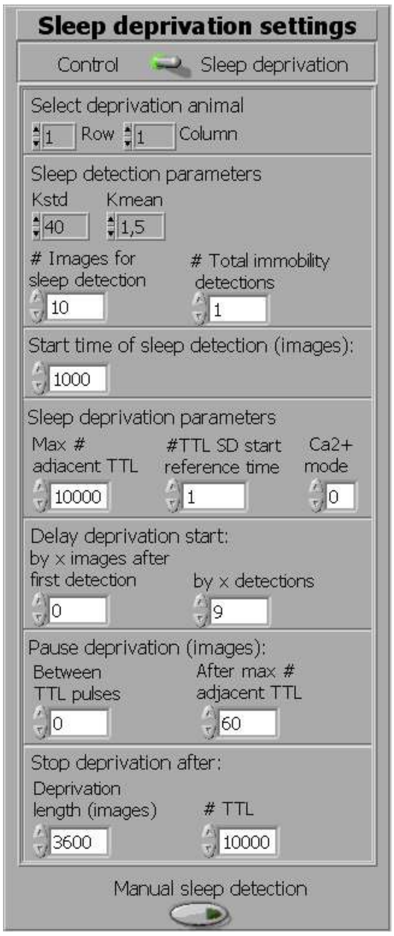

Figure 46: Sleep deprivation settings

Figure 46 shows the sleep deprivation settings

- Mode: Default value (Sleep deprivation), range (Control, Sleep deprivation). The configuration "sleep deprivation" uses automated sleep detection and deprivation. The configuration "control" administers TTL signals according to a list of time points that it reads from a text file.

- Select deprivation animal row/column: Default value (1/1), range (1/1-# animals per row/column).

Sets animal in which sleep gets detected and sleep deprived. The index of row and column is starting with the animal on the down-left. In the displayed example image the animal on the top-left would be annexed with (2,1).

- Sleep detection parameters

k_{std}: Default value (40), range (10-60).
Standard deviation constant for the total immobility sleep detection criterion. Lower
values facilitate sleep recognition in light sleep mutants.

k_{mean}: Default value (1.5), range (1-2).
Mean value constant for the total immobility sleep detection criterion. Higher values facilitate sleep recognition in light sleep mutants.

images for sleep deprivation: Default value (10), range (5-60).
Local time window for which the sleep detection criterion is evaluated. Smaller values facilitate sleep recognition in light sleep mutants, whereas bigger values assure in mutants with ectopic sleep phases or hypo activity that sleep gets not falsely recognized.

(number) Total immobility detections: Default value (2), range (1-10000). Number of times the restrictive sleep detection criterion of total immobility is used. After this number of detections sleep recognition occurs if mobility is lower than a threshold once.

- Start time of sleep detection (images): Default value (1000), range (300-20000).

Time to start evaluation of the sleep detection criterion. Prevents too early false sleep detection. Ideally one sets this value about 2-3 hours prior to the estimated sleep start.

- Sleep deprivation parameters

Max # (number) adjacent TTL: Default value (10000), range (1-10000).
Restricts the number of adjacent TTLs by forcing a pause of deprivation as specified below by the parameter "Pause deprivation after max # adjacent TTL". The number of adjacent TTL being higher than 1 signifies that the animal did not react to the deprivation stimulus.

TTL start SD reference time: Default value (1), range (1-10).
Defines the time point of the x-th TTL as the beginning of sleep deprivation. This is used as the reference time for the deprivation length.

Ca2+ mode: Default value (0), range (0,1,2).

- Delay deprivation start

by x images after first detection: Default value (0), range (0-7200).
Delays the sleep deprivation by at least this amount of time after the first sleep detection. Sleep detection possibly triggering sleep deprivation restarts after this delay has passed.

by x detections: Default value (9), range (0-30).
Do not output a deprivation stimulus for the first x detections.

- Pause deprivation

between TTL pulses: Default value (0), range (0-600).
Forced minimum interval between two TTL pulses.

after max # adjacent TTL: Default value (9),range (0-10).
Forced pause if "max # adjacent TTL", as defined above in sleep deprivation parameters, is reached.

- Stop deprivation after deprivation length: Default value (3600), range (300-10000).

Maximum duration of sleep deprivation. Sleep deprivation start is defined by the time point of the x-th TTL signal, see sleep deprivation parameter "# TTL start SD reference time".

TTL: Default value (10000), range (10-10000). Number of deprivation stimuli delivered after that deprivation gets stopped.

Manual sleep detection: For the time points this button is pressed sleep is detected. This can be used to as replacement of the automated sleep detection or in combination.

Example: Single worm sleep detection with default parameters

General settings

- Select # of animals row/column: (1/1) Sleep deprivation settings

- Select deprivation animal row/column: (1/1)

Example: Multiple worm sleep detection with default parameters

4 animals, deprive the animal on the down-
right. General settings

- Select # of animals row/column:

(2/2) Sleep deprivation settings

- Select deprivation animal row/column: (1/2)

Example: Externally triggered sleep detection with default parameters

Connect externally triggered BNC-cable to Trigger IN of camera

Camera Settings

- Trigger mode: External

Example: Control measurement using predefined stimulus protocol with default parameters

Sleep deprivation settings

- Mode: Control

Create text file containing a list of time points (integer), separated by line breaks.

2.6.10 Optimizing sleep deprivation

Optimizing deprivation
Parameters for sleep deprivation

When to start sleep deprivation

- minimum start time for sleep deprivation
- first sleep detection + x hours
- first sleep detection + x further sleep detections

Design of one stimulus

- pulse length, number of pulses, pause time between pulses

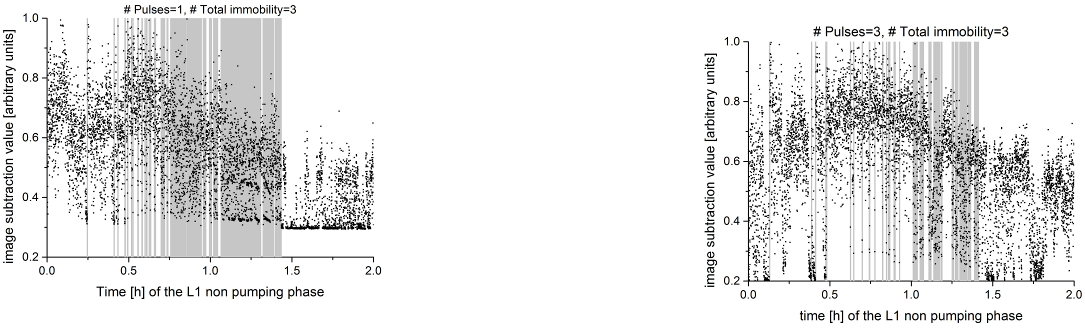

(a) (b) 1

Figure 47: Design of the stimulus. A higher number (#) of consecutive pulses at each sleep detection is more efficient than a single pulse. Shown is the image subtraction value time course for a single animal that receives each time sleep is detected either (a) a single mechanical stimulus or (b) three mechanical stimuli, with an inter stimulus interval of one second. Mechanical stimulation is indicated by gray shading.

Lower the criterion for sleep detection once sleep has been detected for a certain number of times

- number of total immobility detections

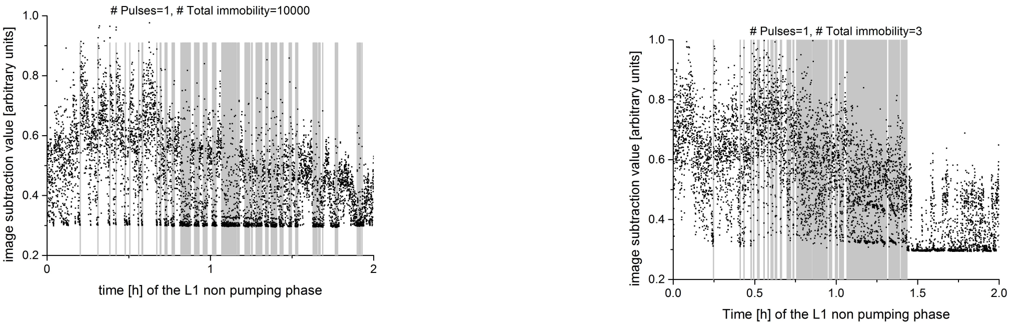

(a) (b) 1

Figure 48: Lowering the sleep detection criterion after sleep has been detected several times yields better sleep deprivation results. Shown is the image subtraction value time course for single animals using either the sleep detection criterion of (a) total immobility only or (b) total immobility for the first three sleep detections followed by low mobility as the sleep detection criterion. Mechanical stimulation is indicated by gray shading.

Limitation of stimulation during sleep deprivation

- forced pause of x seconds between two stimulations

- forced pause of x seconds if a certain number of sequential stimulations is reached

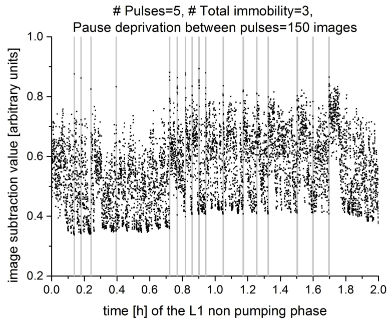

(a)

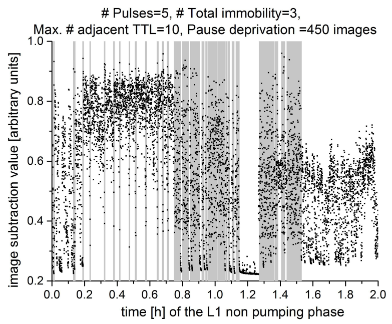

(b)

Figure 49: Limitation of stimulation during sleep deprivation. Shown is the image subtraction value time course for single animals pausing deprivation (a) after each stimulation and (b) if a maximum number of ten adjacent stimulations is reached. Mechanical stimulation is indicated by gray shading.

When to stop sleep deprivation?

- after a certain time of deprivation is reached.
- after a certain number of taps has been delivered.
- if the animal did not react to a certain number of taps.
